# Calcineurin inhibition protects against dopamine toxicity and attenuates behavioral decline in a Parkinson’s disease model

**DOI:** 10.1186/s13578-023-01068-6

**Published:** 2023-08-01

**Authors:** Rupsha Mondal, Chayan Banerjee, Sumangal Nandy, Moumita Roy, Joy Chakraborty

**Affiliations:** 1 CSIR-Indian Institute of Chemical Biology, Kolkata, 700032 India; 2 Academy of Scientific and Innovative Research (AcSIR), Ghaziabad, 201002 India

**Keywords:** Dopamine toxicity, Mitochondrial fragmentation, Calcineurin, Parkinson’s disease, L-DOPA therapy, Dendritic spine

## Abstract

**Background:**

Parkinson’s disease (PD), a highly prevalent neuro-motor disorder is caused due to progressive loss of dopaminergic (DAergic) neurons at substantia nigra region of brain. This leads to depleted dopamine (DA) content at striatum, thus affecting the fine tuning of basal ganglia. In patients, this imbalance is manifested by akinesia, catalepsy and tremor. PD associated behavioral dysfunctions are frequently mitigated by l-DOPA (LD) therapy, a precursor for DA synthesis. Due to progressive neurodegeneration, LD eventually loses applicability in PD. Although DA is cytotoxic, it is unclear whether LD therapy can accelerate PD progression or not. LD itself does not lead to neurodegeneration in vivo, but previous reports demonstrate that LD treatment mediated excess DA can potentiate neurotoxicity when PD associated genetic or epigenetic aberrations are involved. So, minimizing DA toxicity during the therapy is an absolute necessity to halt or slowdown PD progression. The two major contributing factors associated with DA toxicity are: degradation by Monoamine oxidase and DAquinone (DAQ) formation.

**Results:**

Here, we report that apoptotic mitochondrial fragmentation via Calcineurin (CaN)-DRP1 axis is a common downstream event for both these initial cues, inhibiting which can protect cells from DA toxicity comprehensively. No protective effect is observed, in terms of cell survival when only PxIxIT domain of CaN is obstructed, demonstrating the importance to block DRP1-CaN axis specifically. Further, evaluation of the impact of DA exposure on PD progression in a mice model reveal that LD mediated behavioral recovery diminishes with time, mostly because of continued DAergic cell death and dendritic spine loss at striatum. CaN inhibition, alone or in combination with LD, offer long term behavioral protection. This protective effect is mediated specifically by hindering CaN-DRP1 axis, whereas inhibiting interaction between CaN and other substrates, including proteins involved in neuro-inflammation, remained ineffective when LD is co-administered.

**Conclusions:**

In this study, we conclude that DA toxicity can be circumvented by CaN inhibition and it can mitigate PD related behavioral aberrations by protecting neuronal architecture at striatum. We propose that CaN inhibitors might extend the therapeutic efficacy of LD treatment.

**Supplementary Information:**

The online version contains supplementary material available at 10.1186/s13578-023-01068-6.

## Background

Parkinson’s disease (PD) is one of the highly prevalent neurodegenerative disorders, primarily caused by progressive loss of dopaminergic (DAergic) neurons at substantia nigra (SN) region of brain. Although short term symptomatic relief is well achievable by administering dopamine (DA) precursor l-DOPA (LD), PD does not have a cure yet. This is mainly due to unavailability of a common druggable target which can modulate multiple aspects of PD progression. Among the opinions which partially explain why discrete regions of brain suffer heavy neuronal loss in PD, DA toxicity is one of the highly studied factors [1–5]. There are different insights which help to understand the detrimental effects of DA on neurons and most of them might cumulatively contribute to the disease progression. Two of the major justifications given in this regard involve: (i) free radical generation during Monoamine oxidase (MAO) mediated DA degradation [6] and (ii) mitochondrial stress due to the formation of DAquinones (DAQ) [3, 4]. In literature, it is evident that both the pathways may affect mitochondrial homeostasis and follow similar route towards cell death [7–9]. Although mitochondrial dysfunction is a well-established fact in PD [10, 11], extent of contribution by DA in this regard is still elusive. Further, it is not clear whether DAQ or MAO mediated cellular toxicity share a common point, which can be controlled to mitigate DA toxicity more comprehensively. Identifying and characterizing such a shared event might have broad implication to manage elevated DA associated toxicity, and thus extend the efficacy of LD therapy. We hypothesize that initial pre-apoptotic conditioning of mitochondria could be one such event, constraining which may delay neuronal death.

Due to the impact of DA on cell survival, it is argued that LD therapy might accelerate PD progression. However, from clinical studies it is not clearly evident. Although LD therapy might not have any detrimental effect on behavior, SPECT neuroimaging studies kept the hypothesis of accelerated neurodegeneration open [12, 13]. In this regard, it is conceivable that unregulated high DA production can intensify stress on declining neuronal population and affect mitochondrial physiology further.. Given the challenge of determining a definitive choice between possible accelerated neuron loss and guaranteed short-term behavioral relief, opting for LD alone therapy remains a viable initial approach to manage Parkinson's disease. Lack of neurodegeneration after high dosage of LD in different animal models further advocated that it may not cause neurodegeneration by itself [14, 15]. However, DA can potentiate neuronal loss in animal models where mitochondrial functioning is compromised. Study by Burbulla et al. [16] demonstrated that LD or PD associated DJ1 mutation alone did not lead to DAergic neuron loss in vivo, but elevated levels of DA in DJ1 mutant mice could trigger the death cascade. Interestingly, this effect of DA was evaded by antioxidants and Calcineurin (CaN) inhibition. So, it appears that elevated DA itself may not demonstrate neurotoxic effects in vivo (at least in short term). However, when mitochondrial homeostasis is compromised in diseased states, LD treatment might have exponential effects on neuronal mortality.

Protective role of antioxidants in different PD models is known for long, however, they do not qualify for stand-alone therapy against PD since it is difficult to achieve or maintain their active concentrations at a physiological level [17–21]. In this regard, managing the other aspects (such as CaN inhibition) can be considered for synergistic therapy development to expand the LD responsive period in PD patients. CaN as a phosphatase is at the juncture of many physiological pathways, and apoptotic mitochondrial fission via DRP1 activation is one such event [22–24]. Effect of CaN inhibition on PD associated behavioral complexities or DA neuronal loss in presence of LD is not evaluated yet. In this study we used neuronal cell line to evaluate the protective effect of CaN modulation against DA toxicity. Further, we utilized 1-methyl-4-phenyl-1,2,3,6-tetrahydropyridine (MPTP) induced mice model of sporadic PD to monitor the effect of chronic DA supplementation (in presence or absence of CaN inhibitor) on behavior, DA levels and neuroanatomy. We found that CaN inhibition can mitigate DA induced cell death by inhibiting DRP1 mediated apoptotic mitochondrial fragmentation. Further, CaN inhibitor alone or in combination with LD, offered better behavioral outcomes in the PD model when compared to LD alone. CaN-DRP1 axis specifically appeared vital for such protections in behavior and neuroanatomy.

## Results

### Monoamine oxidase (MAO) and DAquinone (DAQ) contribute to dopamine (DA) induced cell death

**Fig. 1:**
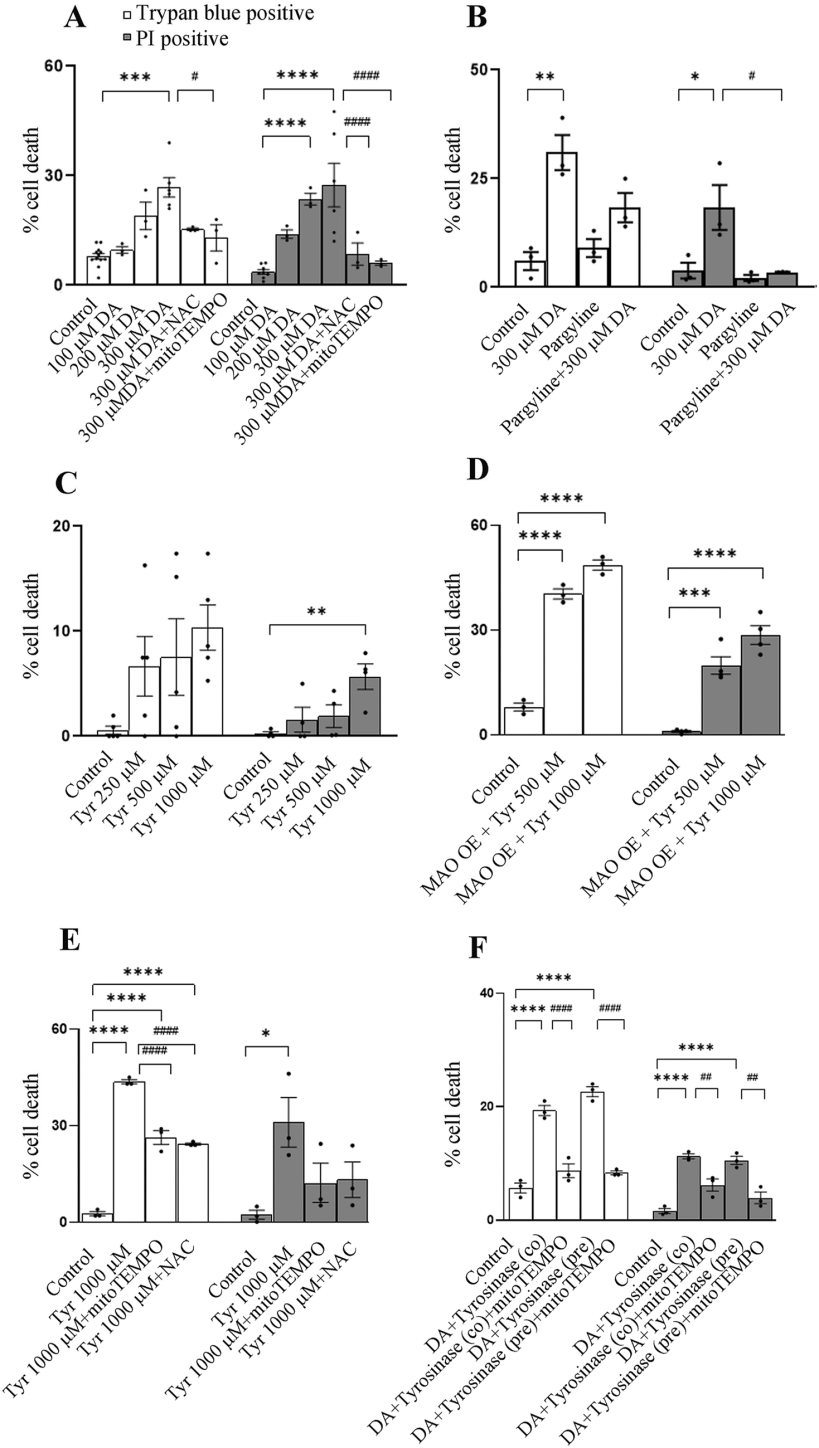
Dopamine, tyramine or DAquinone leads to cell death. **A**, **B** Bar graphs show cell death after 24 h, as quantified by trypan blue or propidium iodide (PI) positive cells. SH-SY5Y cells are treated with different concentrations of dopamine (DA). *N*-acetyl cysteine (NAC)/mitoTEMPO/pargyline are administered 30 min before DA treatment. *P ≤ 0.05, **P ≤ 0.01, ***P ≤ 0.001, ****P ≤ 0.0001 when compared to control group. #P ≤ 0.05, ###P ≤ 0.001, ####P ≤ 0.0001 when compared to the 300 µM DA treated group. **C** Cell death is measured in SH-SY5Y cells, as mentioned in **A**, after 24 h of tyramine (Tyr) treatment. **P ≤ 0.01 when compared to control group. **D** Monoamine oxidase A is overexpressed in SH-SY5Y cells and tyramine (Tyr) in mentioned concentration is treated for 24 h. Bar graphs represent cell as in **A**. ***P ≤ 0.001, ****P ≤ 0.0001 as compared to control. **E** SH-SY5Y cells are treated with tyramine (Tyr) for 48 h. NAC or mitoTEMPO is treated 30 min before Tyr treatment. Cell death is measured as mentioned above. **P ≤ 0.01, ***P ≤ 0.001 when compared to control group. #P ≤ 0.05 when compared to the 1000 µM Tyr treated group. **F** Cells are treated with DA (300 µM) and Tyrosinase (co: co treated) for 24 h. Alternately DA and Tyrosinase is incubated beforehand (pre: pre-incubation) and then administered to the cells. In the relevant groups mitoTEMPO is administered 30 min before the treatment. Cell death is measured as mentioned in **A**. ****P ≤ 0.0001 when compared to control group., ^##^P ≤ 0.01, ^###^P ≤ 0.0001 when compared to the respective 300 µM DA + Tyrosinase treated group. Bar graphs represent mean ± SEM. N = at least 3 for each group. P values are calculated by one way ANOVA followed by Tukey’s/Dunnett’s multiple comparison test, when compared between mean of each group or mean of control group, respectively

To measure DA mediated cell death, we quantified both trypan blue and PI positive cells after 24 h of DA treatment. Cell death in SH-SY5Y cell line is evident within 24 h of 200 or 300 µM DA treatment (Fig. 1A). In order to investigate the role of oxidative stress in the DA-induced cell death, we incorporated *N*-acetyl cysteine (NAC) and mitoTEMPO into the treatment groups. NAC enhances the levels of l-cysteine, a precursor of antioxidant glutathione. MitoTEMPO, on the other hand, is an antioxidant that primarily accumulates within mitochondria. 300 µM DA induced cell death is partially attenuated by NAC and mitoTEMPO (Fig. 1B). Interestingly pargyline, an irreversible MAO inhibitor also demonstrated the same effect (Fig. 1B). This might suggest the involvement of reactive oxygen species (ROS) generation via MAO activity as a cause for DA induced cell death. To evaluate if any MAO substrate can lead to similar cell death, we treated these cells with a well-known MAO substrate (primarily MAO A)—tyramine (Tyr). Tyr, even at a dose of 1 mM induced only ~ 5% cell death after 24 h (Fig. 1C). However, administering Tyr to MAO A overexpressing SH-SY5Y cells heighten cell mortality (Fig. 1D; Additional file 1: Fig. S1). Extending treatment period for un-transfected cells up to 48 h also enhance Tyr toxicity, which can be attenuated by NAC or mitoTEMPO treatment (Fig. 1E).

Auto-oxidation or enzymatic conversion can result in the formation of DAQ from DA. Tyrosinase oxidizes DA (leading to the formation of DAQ) without the production of free radicals [5, 9, 25]. To determine the effect of DAQ alone on cell survival, one group of cells were treated with DA and Tyrosinase simultaneously. In another group, we first incubated DA with Tyrosinase for the duration of thirty minutes, and then administered the entire reaction mixture to the cells. Both the treatment groups demonstrated similar toxicity and are comparable to cell death caused by DA alone (300 µM). Cell mortality due to both these treatments is attenuated by mitoTEMPO (Fig. 1F). Tyrosinase alone (without DA) does not induce cell death within this time period (data not shown).

### Dopamine (DA), Tyramine (Tyr) or DAquinone (DAQ) treatment leads to mitochondrial fragmentation

**Fig. 2:**
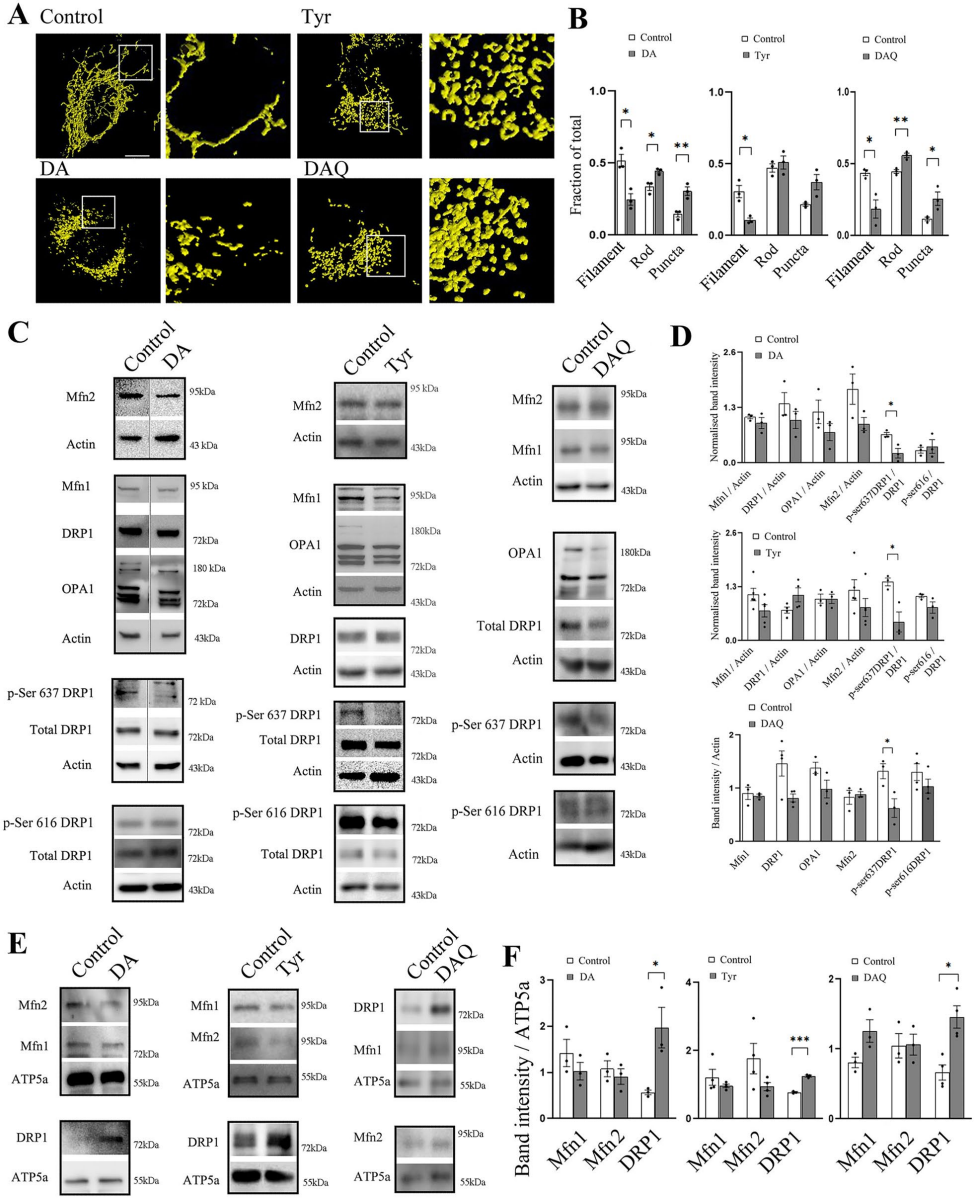
Dopamine, Tyramine, or DA-quinone treatment induce mitochondrial fragmentation, decrease p-Ser637 DRP1 and increase DRP1 mitochondrial localization.** A** SH-SY5Y cells are treated with DA (200 µM), Tyramine (1 mM) or DAQ (200 µM co-incubation with Tyrosinase) for 16 h and mitochondria is visualized by immunostaining for ATP5a. Scale bar 10 µm. **B** Mitochondrial morphology is analyzed after the treatment as mentioned above, and mean proportion of filamentous, rod shaped or punctate mitochondria is represented in the graphs. At least 30 cells are analyzed from 3 independent experiments for each group. **C** SH-SY5Y cells are treated as mentioned in A and total cell lysate is subjected to SDS PAGE followed by immunoblotting for the mentioned proteins. Immunoblots are representative of at least 3 different experiments. **D** Mean normalized band intensities of the mentioned proteins in **C** are represented as bar graphs. **E** SH-SY5Y cells are treated as mentioned in **A** and protein lysate from mitochondria is subjected to SDS PAGE followed by immunoblotting for the mentioned proteins. Immunoblots are representative of at least 3 different experiments. **F** Mean normalized band intensities of the mentioned proteins in E are represented as bar graphs. Bar graphs represent mean ± SEM. N = at least 3 for each group. P values are calculated by Student’s t test. *P ≤ 0.05, **P ≤ 0.01 and ***P ≤ 0.001 compared to control group

Mitochondrial fission/fragmentation is often a precondition for cell death [22, 26–28]. So, next we evaluated mitochondrial morphology after 16 h of DA (200 µM), DAQ (200 µM DA + Tyrosinase) or Tyr (1 mM) treatment. SH-SY5Y cells exhibit profound loss of filamentous mitochondria and increase in fragmented or punctate mitochondria in all the three treatment groups (Fig. 2A, B). Mitochondrial fusion is mainly controlled by Optic atrophy 1 (OPA1, a GTPase responsible for fusion of inner mitochondrial membrane), Mitofusin 1 and Mitofusin 2 (Mfn1 and Mfn2 respectively, both responsible for outer mitochondrial membrane fusion). Mfn2 also tether the organelle with endoplasmic reticulum; which can mark the site of mitochondrial fission. Reduced levels or altered functioning of these proteins lead to spheroid like small mitochondria [22, 29]. Total OPA1, Mfn1and Mfn2 levels remained unaltered after DA/Tyr/DAQ treatment (Fig. 2C, D). A trend towards decrease is noticeable in total DRP1 levels after DAQ treatment (Fig. 2C, D).

On the other hand, mitochondrial fission is primarily regulated by GTPase Dynamine related protein 1 (DRP1). Reduced level of DRP1 leads to mitochondrial elongation whereas increased mitochondrial translocation of DRP1 fragments the organelle. Different post translational modifications regulate mitochondrial translocation of DRP1 and one of the highly studied modifications is phosphorylation at different sites [29]. The two major sites are at DRP1 Ser637 and Ser616. Phosphorylation of DRP1 at Ser616 or Ser637 (p-Ser637 and p-Ser616, respectively) have contrasting effects, and therefore recruitment of DRP1 cannot be determined by either one alone [30]. Instead, the overall balance between these phosphorylation events can determine the shape of the organelle [31, 32]. Reversible phosphorylation of DRP1 at Ser637 blocks fission whereas de-phosphorylation by CaN leads to its mitochondrial translocation [32]. Phosphorylation at Ser616 enhances DRP1 localization on mitochondria and thus fission [33]. Excessive mitochondrial fission is often followed by apoptosis [22, 24, 27]. Although p-Ser616DRP1 levels remain unaltered after DA /Tyr/DAQ treatment, interestingly, p-Ser637DRP1 levels are found to be reduced. Reduction in p-Ser637DRP1 level indicates translocation of DRP1 to mitochondria. To confirm mitochondrial translocation, we isolated mitochondria rich fraction after treatment and compared Mfn1, Mfn2 and DRP1 protein levels (Fig. 2E, F). Level of Mfn1 and Mfn2 remain unchanged in mitochondrial fraction after DA/Tyr/DAQ treatment; however, as expected DRP1 levels are significantly increased.

### Dopamine-induced mitochondrial fragmentation via Monoamine oxidase-Calcineurin-DRP1 axis leads to Cytochrome c re-localization and cell death

**Fig. 3:**
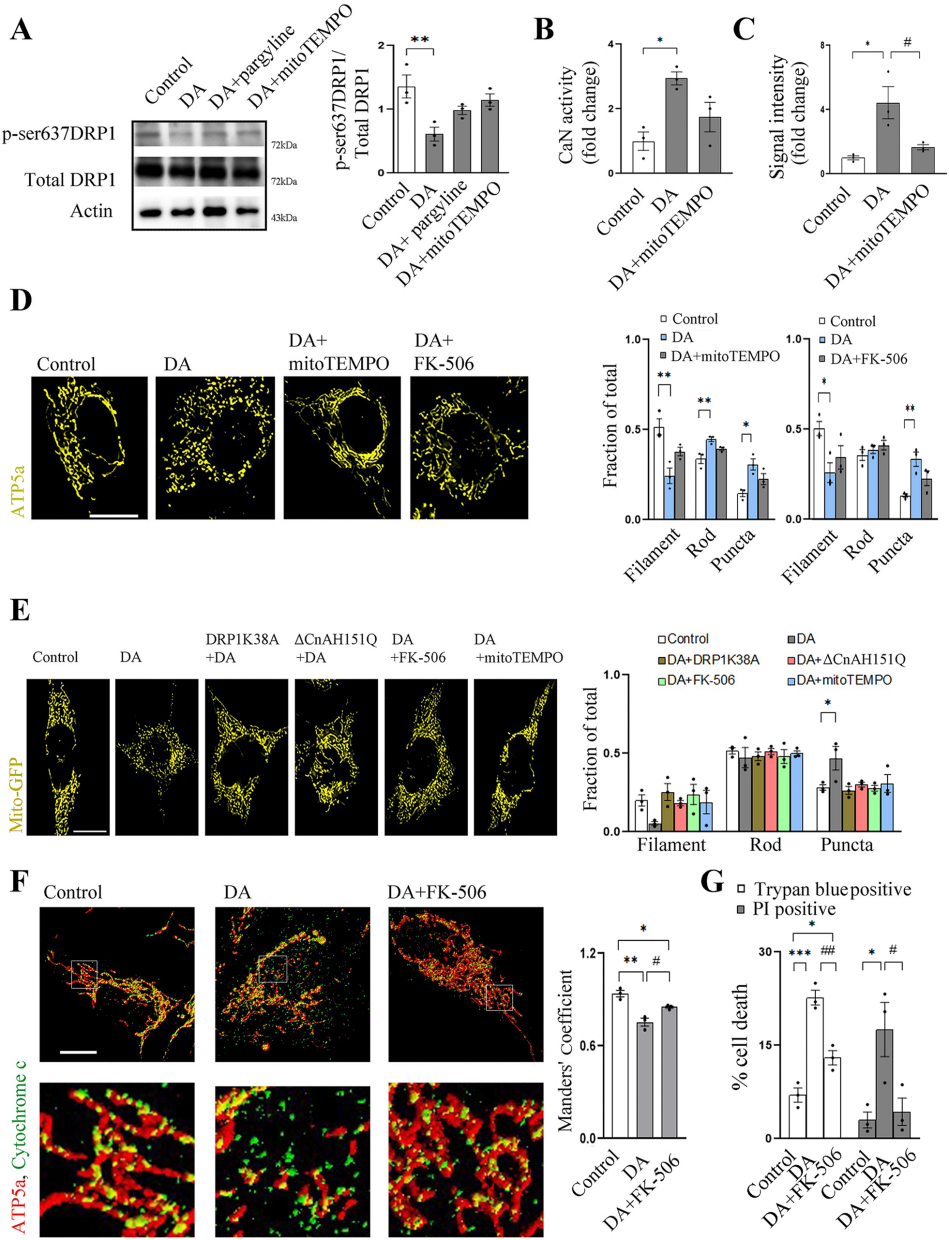
Dopamine induced p-Ser637DRP1 depletion, mitochondrial fragmentation and apoptotic Cytochrome c re-localization is attenuated by Calcineurin inhibition. **A** SH-SY5Y cells are treated with DA (200 µM) or as mentioned for 16 h and total cell lysate is subjected to SDS PAGE followed by immunoblotting for the mentioned proteins. Bar graphs represent mean intensity value of p-Ser637DRP1 normalized by total DRP1. Immunoblots are representative of at least 3 different experiments. **P ≤ 0.05 as compared to control, one way ANOVA followed by Tukey’s multiple comparison test. **B** After the mentioned treatment for 16 h, Calcineurin (CaN) activity from the SH-SY5Y cell lysate is measured and the mean activity is represented. *P ≤ 0.05. **C** SH-SY5Y cells are treated for 16 h and cytosolic free Ca^2+^ is measured by Fluo-4-AM intensity. Mean intensity of each group is represented as bar graph. *P ≤ 0.05 as compared to control, #P ≤ 0.05 when compared to DA treated group. **D** After treatment, as mentioned, mitochondrial morphology of SH-SY5Y cells is analyzed from the captured images. Images are representative of 3 different experiments and at least 30 cells were analyzed. Scale bar 10 µm. Mitochondrial morphology is classified as mentioned earlier and mean proportion of filamentous, rod shaped or punctate mitochondria is represented in the bar graphs. *P ≤ 0.05, **P ≤ 0.01 as compared to control. **E** Control or MEF cells expressing DRP1K38A/dominant negative Calcineurin (ΔCnAH151Q) are treated as mentioned, for 16 h and mitochondria are imaged. Mitochondrial classification and quantification are done as mentioned above. *P ≤ 0.05 as compared to control. Scale bar 10 µm. **F** SH-SY5Y cells are treated with DA or DA + FK-506 for 16 h. Fixed cells are stained for ATP5a and Cytochrome c. Co-localization is quantified by measuring Mander’s coefficient and mean values are represented as bar graph. At least 30 cells were analyzed from 3 independent experiments. *P ≤ 0.05, **P ≤ 0.01 as compared to control, #P ≤ 0.05 as compared to DA treated group. **G** SH-SY5Y cell death is analyzed by propidium iodide (PI) or trypan blue staining, after DA or DA + FK-506 treatment for 24 h. *P ≤ 0.05, ***P ≤ 0.001when compared to control group; #P ≤ 0.05 and ##P ≤ 0.01 when compared to DA treated group. Bar graphs represent mean ± SEM. N = at least 3 for each group. P values are calculated by one way ANOVA followed by Tukey’s multiple comparison test unless mentioned otherwise

To determine whether inhibiting MAO or mitochondrial ROS can attenuate DA mediated decrease in p-Ser637DRP1 levels, we pretreated the cells with pargyline and mitoTEMPO. p-Ser637DRP1 level is partially rescued by both pargyline and mitoTEMPO (Fig. 3A). As stated before, DRP1 phosphorylation at Ser637 is controlled by CaN [32]. Analysis of CaN activity indeed shows an elevation after DA treatment (Fig. 3B). Increase in CaN activity is not detected when the cells are pretreated with mitoTEMPO (Fig. 3B). This increase in CaN activity and attenuation by ROS scavenging can be correlated with the rise in cytosolic Ca^2+^ levels after 16 h of DA treatment, as measured by Fluo4 AM intensity (Fig. 3C). As a follow up, we ascertained whether scavenging mitochondrial ROS or inhibiting CaN can protect DA induced aberrations in mitochondrial morphology. We found that mitoTEMPO treatment could decrease DA induced mitochondrial fragmentation (Fig. 3D). For inhibiting CaN we used two different inhibitors separately—cyclosporine A (CsA) and FK-506. CsA or FK-506 (in complex with Cyclophilin or FKBP, respectively) can equally inhibit CaN phosphatase activity [34]. Both the inhibitor protected the cell from DA induced mitochondrial fragmentation (Additional file 2: Fig. S2A; Fig. 3D). However, CsA can also inhibit mitochondrial permeability transition pore and thus block apoptosis, independent of CaN activity [35, 36]. Due to higher specificity towards CaN, we utilized FK-506 for further experiments. For further validation, we expressed dominant negative mutant of DRP1 (DRP1K38A) or CaN (ΔCnAH151Q) in MEF cells and treated with DA for 16 h. Analysis of the mitochondrial morphology demonstrates that both CaN activity and DRP1 is required for DA induced mitochondrial fragmentation (Fig. 3E). Pargyline and mitoTEMPO treatment also attenuated DA induced mitochondrial morphology aberrations in MEF cells (Fig. 3E). FK-506 or mitoTEMPO treatment can also rescue Tyr or DAQ induced mitochondrial fragmentation after 16 h treatment (Additional file 2: Fig. S2B, C).

During prolonged stress, mitochondrial fission often precedes apoptosis [27]. After fragmentation mitochondria loses membrane potential and release internal components to cytosol through mitochondrial permeability transition pore [26, 37]. Among these components is—Cytochrome * c* which in cytosol can form apoptosome complex. This complex can activate different Caspases and culminate to cell death [38]. Increased cytosolic Cytochrome *c* is detected in SH-SY5Y cells after DA treatment, which is prevented by FK-506 treatment (Fig. 3F). This is also reflected on cell survival, where FK-506 treatment could partially attenuate DA induced cell death (Fig. 3G). Partial protection against DA induced cell death is also observed in DRP1 KO MEF cells (Additional file 3: Fig. S3).

The binding sites of CaN on its substrates have been partially determined by extensively using transcription factor NFAT as a representative model. There are two well-recognized binding sites of CaN on NFAT: PxIxIT (located near the N-terminal region) and LxVP (located near the C-terminal region). Among the NFAT protein family members and other CaN substrates (such as TRESK, Crz1, Slm1, and Hph1), highly conserved PxIxIT serves as the primary site of interaction. Conversely, LxVP has a weaker binding affinity with CaN, but it is positioned closer to the active site. However, sequence variations within these conserved positions are possible and comparative affinity of CaN towards such proteins is a matter of exploration [39, 40]. Although DRP1 is regulated by CaN under stress, it lacks a conserved PxIxIT consensus motif. By employing VIVIT peptide, we blocked the docking site of CaN at PxIxIT. If cell death induced by DA is primarily driven by the interaction of CaN with its other partners, inhibiting this site would provide protection against cell death. Interestingly, blockade of PxIxIT domain, by the treatment of VIVIT peptide [40] or INCA -6 (an inhibitor of NFAT-CaN binding by blocking PxIxIT site) [41] cannot block DA induced mitochondrial fragmentation or cell death (Additional file 4: Fig. S4A, B), which minimize the possible major involvements of PxIxIT containing CaN substrate in the current context.

### FK-506, alone or in combination with l-DOPA (LD) rescue behavioral decline in MPTP induced mice model of Parkinson’s disease

To assess the impact of increased DA, in vivo; we selected the highest effective dose which offers significant improvement of motor activity in PD mice and does not cause any hyperactivity [42, 43]. 10 mg/kg LD increased CaN activity at striatum significantly, whereas a lower dose (5 mg/kg) failed to do so (Additional file 5: Fig. S5A). FK-506 (1 mg/kg, gavage) demonstrated significant inhibition in striatal CaN activity (Additional file 5: Fig. S5B). Increase in CaN activity is also reflected at p-Ser637 DRP1 levels, which decrease after 5 days of LD (10 mg/kg) treatment (Additional file 5: Fig S5C). This dose does not show any marked apoptotic event at striatum even after 12 days of administration, as measured by TUNEL positive cells (Additional file 5: Fig. S5D). DAergic cell bodies at SN or distal projections at striatum also remain unaltered (Additional file 5: Fig. S5E, F). Spine density of the striatal medium spiny neurons is unaffected; however, a shift towards increased immature (budding) spines after 12 days of LD (10 mg/kg) treatment is noticeable. Interestingly, FK-506 treatment (1 mg/kg) demonstrated higher number of mature spines (Additional file 5: Fig. S5G, H).

**Fig. 4:**
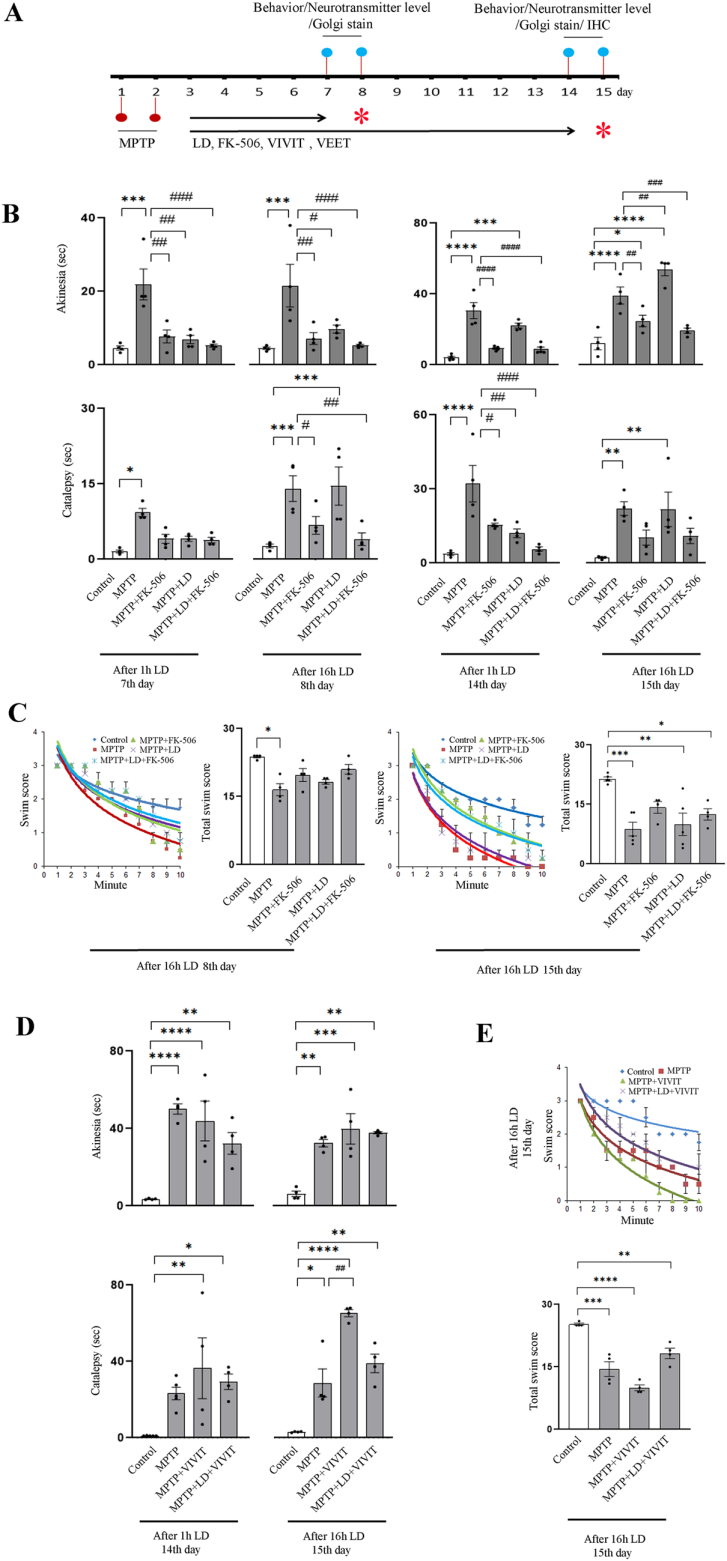
Calcineurin inhibition protects against MPTP induced behavioral deficiency in presence/absence of L-DOPA while VIVIT peptide fails to offer any. **A** Treatment paradigm for MPTP induced Parkinson’s disease model generation. MPTP (30 mg/kg) is treated on day 1 and 2 (16 h apart). From 3rd day the mentioned treatments are initiated, alone or in combination. Behavior is evaluated on 7th, 8th, 14th and 15th day. On 8th and 15th day, behavioral assays are done after 16 h of the last treatment. Neurotransmitter level analysis and histochemistry are done on either 8th or 15th day. [*] in red denotes the day in which animals are sacrificed. **B** Akinesia and catalepsy on the mentioned days for the treatment groups are evaluated. At least 4 animals are analyzed for each experiment. *P ≤ 0.05, ***P ≤ 0.001, ****P ≤ 0.0001 when compared to control group; ^#^P ≤ 0.05, ^##^P ≤ 0.01,^###^P ≤ 0.001,^####^P ≤ 0.0001 when compared to MPTP treated group. P values are calculated by two way ANOVA followed by Tukey’s multiple comparison test. **C** Trajectories from the mean swim score (± SEM) for the mentioned time is plotted and the cumulative score for 10 min, for each group on 8th and 15th day is represented. At least 4 animals are taken for the analysis. *P ≤ 0.05, **P ≤ 0.01,***P ≤ 0.001 when compared to the control group. P values are calculated by one way ANOVA followed by Tukey’s multiple comparison test. **D** Akinesia and catalepsy are evaluated on the mentioned days for the treatment groups. At least 4 animals are taken for the evaluation. *P ≤ 0.05, **P ≤ 0.01,***P ≤ 0.001, ****P ≤ 0.0001 when compared to control group; ^##^P ≤ 0.01 when compared to MPTP treated group. P values are calculated by two way ANOVA followed by Dunnetts multiple comparison test. **E** Trajectories from the average swim score (± SEM) for the mentioned time is plotted and the cumulative score for 10 min, for each group on 15th day is represented. At least 4 animals are taken for the analysis. **P ≤ 0.01,***P ≤ 0.001 and **** P ≤ 0.0001 when compared to the control group. P values are calculated by one way ANOVA followed by Dunnett’s multiple comparison test. Bar graphs represent mean ± SEM

Next, we wanted to determine whether this dose of LD (10 mg/kg) could influence DAergic neuronal survival as well as behavior in a diseased state. We developed MPTP induced mice model of sporadic PD [44, 45]. MPTP undergoes bio-transformation in the brain by MAO, resulting in the formation of MPP^+^. This compound selectively accumulates within DAergic neurons. The toxicity of MPP^+^ is attributed to its capacity to inhibit electron transport chain complex I [46]. It was previously demonstrated that two doses of MPTP (30 mg/kg; i.p. 16 h apart) is sufficient to induce the disorder [45, 47]. To follow the progression of the disease, we sacrificed the animals on 8th or 15th day after first MPTP injection. LD treatment was started from the 3rd day and continued till 14th day (Fig. 4A). Interestingly, we found that MPTP or MPTP + LD treatment increased striatal CaN activity on 8th day, (Additional file 6: Fig. S6A). This effect is reflected on striatal p-Ser637DRP1 on 8th day (Additional file 6: Fig. S6B).

For behavioral assessment, we selected two of the classical PD related behavioral complexities: akinesia and catalepsy. These two behaviors do not involve any learning ability or strongly forced motor activity. Additionally, we also included swim test on 8th and 15th day to determine the behavioral outcome in response to intense stimuli. To evaluate the behavioral outcomes, we selected 4 time points: 7th day (after 1 h of LD), 8th day (after 16 h of LD), 14th day (after 1 h of LD) and 15th day (after 16 h of LD; Fig. 4A). On 7th day, akinesia is clearly visible in MPTP treated mice; whereas no alterations compared to control are noticed in LD, FK-506 or LD + FK-506 treated groups (Fig. 4B). The same is evident in catalepsy test (Fig. 4B). This behavioral protection, in terms of akinesia persists even after 16 h of LD treatment in the LD alone or LD + FK-506 treated PD mice on 8th day. Interestingly, FK-506 group also continue to demonstrate unaltered akinesia on 8th day (Fig. 4B). Catalepsy remains unaltered on 8th day (16 h after LD treatment) when compared to control in LD + FK-506 or FK-506 group, whereas LD alone group demonstrate increased latency (Fig. 4B).

The protective effect of LD on akinesia in PD mice is absent on 14th day (Fig. 4B). However, akinesia is unaltered in FK-506 or LD + FK-506 group, compared to control. This trend remained unchanged on 15th day as well (Fig. 4B). Interestingly, catalepsy is significantly attenuated by FK-506, LD + FK-506 or LD treatment, compared to the MPTP treated mice on 14th day. On 15th day, high variability in cataleptic behavior is observed in LD alone group, whereas partial rescue in cataleptic behavior is observed LD + FK-506 or FK-506 group (Fig. 4B). Significant deficiency in swim ability on 8th day is noticeable in MPTP treated mice, which remain unaltered in other treatment groups, as compared to control (Fig. 4C). On 15th day, swim inability is visible in MPTP, LD alone or LD + FK-506 treated PD mice (Fig. 4C).

Interestingly, VIVIT peptide administration (0.5 mg/kg) upto 14th day (alone or in combination with LD) could not protect akinesia, catalepsy (Fig. 4D) or swim activity (Fig. 4E) induced by MPTP on 15th day. VEET peptide (nonspecific peptide) also demonstrated inability to rescue MPTP induced decline in behavior (Additional file 7: Fig S7A, B).

### FK-506 treatment attenuates behavioral decline in PD mice by protecting medium spiny neuronal architecture at striatum

**Fig. 5:**
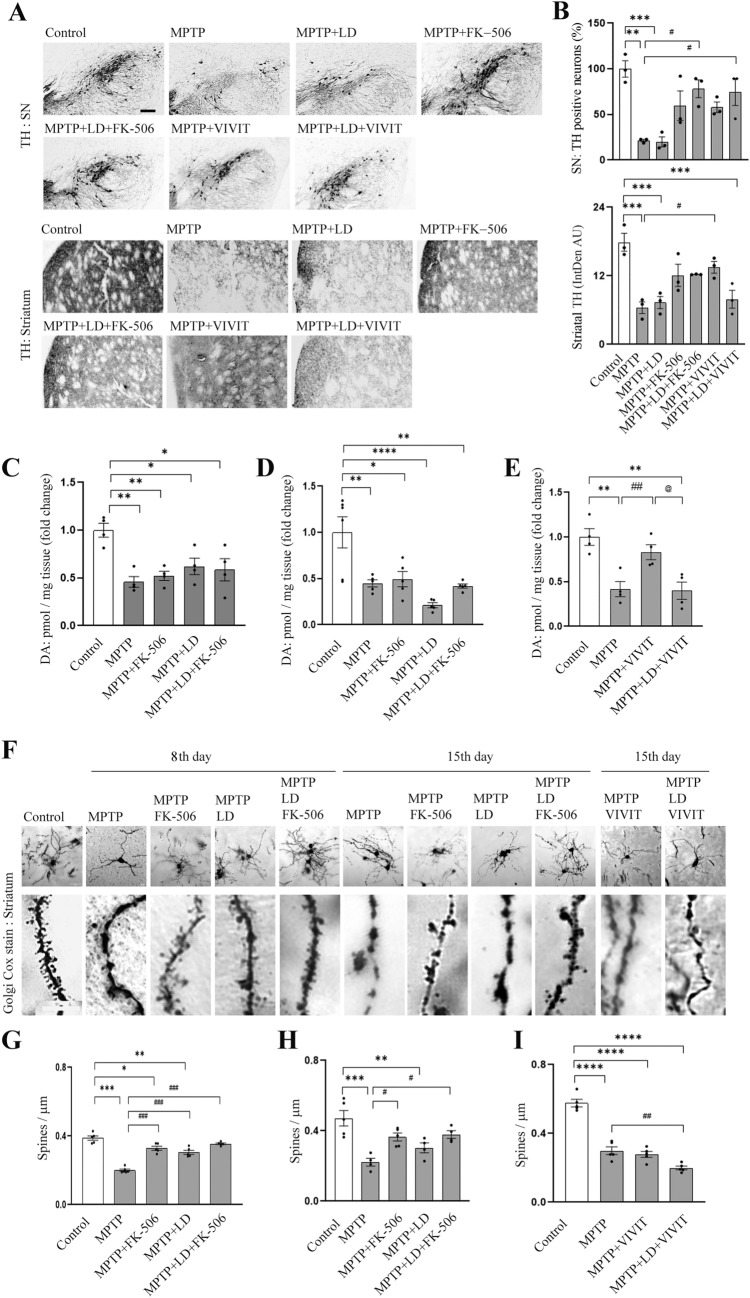
Calcineurin inhibition protects against MPTP induced depletion of TH positive neurons at SN and dendritic spine density at striatum, while VIVIT peptide fails to safeguard the later. **A** Immunohistochemical staining for Tyrosine hydroxylase (TH) is performed after 14 days of treatment for substantia nigra (SN) and striatal cryosections (20 µm). Scale bar—100 µm. **B** TH positive neuronal cell bodies at SN or striatal projections are measured. N = 3 (**C**–**E**) After the treatment period striatal dopamine (DA) is quantified by HPLC method and represented as pmol/mg tissue. At least 4 animal striatum is utilized for the analysis. (**C**, **D**) denote changes in DA levels on 8th and 15th day respectively, while (**E**) shows the changes on 15th day. *P ≤ 0.05, **P ≤ 0.01, ****P ≤ 0.0001 when compared to the control group, ^##^P ≤ 0.01 when compared to the MPTP treated group. ^@^P ≤ 0.05 when compared to the MPTP + VIVIT treated group. P values are calculated by one way ANOVA followed by Tukey’s multiple comparison test. **F** Striatal sections (60 µm) are stained by Golgi-Cox method and representative images are provided. The full images of the neurons are presented in the inset. Scale bar 20 µm for inset image and 10 µm for the enlarged dendritic portion. **G**–**I** Spine number in entire length on individual dendritic projections from each neuron is counted and the mean number of spines are represented for each treatment group on the 8th (**G**) and 15th (**H**, **I**) day. At least 5 animal striatum and 4–5 neurons from each striatum are considered for the analysis. *P ≤ 0.05, **P ≤ 0.01, ***P ≤ 0.001, ****P ≤ 0.0001 when compared to control group; #P ≤ 0.05, ##P ≤ 0.01, ###P ≤ 0.001, when compared to MPTP treated group. Bar graphs represent mean ± SEM. P values are calculated by one way ANOVA followed by Tukey’s multiple comparison test

To correlate MPTP induced PD associated behavioral outcomes with neuronal survival, neurotransmitter and neuroanatomical changes, we further analyzed two DA rich regions of brain: SN and striatum. FK-506 alone or in combination with LD protected TH positive neurons at SN (Fig. 5A, B). Although VIVIT could not protect behavioral outcomes (Fig. 4D, E), it is effective against MPTP induced decline in DAergic neuronal number at SN (Fig. 5A, B), whereas VEET treatment fail to do so (Additional file 7: Fig. S7D). Less DAergic neuronal terminals at striatum is observed in MPTP and MPTP + LD treated groups, which is attenuated to some extent by FK-506 or VIVIT treatment (Fig. 5B)., Although the protective effect of VIVIT in MPTP + LD + VIVIT group is visible at SN, striatal TH staining is reduced in this group (Fig. 5A, B).

Decline in MPTP induced striatal DA level is not protected in LD/FK-506 or LD + FK-506 group on 8th or 15th day (Fig. 5C, D, respectively). Interestingly, VIVIT alone can maintain striatal DA levels after MPTP treatment on 15th day; however, the effect diminishes in presence of LD (Fig. 5E). VEET peptide treatment cannot protect MPTP induced decline in DA level or TH positive neurons at SN (Additional file 7: Fig. S7C, D).

Next, we evaluated the status of neuronal morphology at striatum. MPTP treatment led to heavy loss of dendritic spines in striatal medium spiny neurons on 8th day, which persisted on 15th day as well (Fig. 5F, G, H). On 8th day spine density is protected by FK-506 and LD (alone or together), but on 15th day this protective effect is only visible in FK-506 and LD + FK-506 treatment groups (Fig. 5F, H). VIVIT or VEET treatment could not protect MPTP induced reduction in spine density on 15th day (Fig. 5F, I; Additional file 7: Fig. S7E). LD + VIVIT treatment in PD mice demonstrated significantly decreased spines when compared to the MPTP alone group.

## Discussion

Whether or not LD treatment accelerates PD progression in patients is controversial [12, 13, 48], but it is a fact that LD is the most successful and well-practiced therapy currently available to address PD associated behavioral complexities. However, long term LD usage has adverse effects like dyskinesia [49] and thus a high proportion of studies remain oriented towards managing those complexities. MAO inhibitor and DA receptor agonist are currently the options to reduce LD dosage, at least during initial stages, but none of these address DA toxicity broadly [50–53]. In this study we found that Tyr, DAQ or DA leads to CaN-DRP1-mediated mitochondrial fragmentation which eventually lead to cell death in vitro. Thus, implication of CaN inhibition is assessed in vivo to mitigate PD associated behavioral decline and neurodegeneration, in presence or absence of LD. Inhibiting specifically CaN-DRP1 axis, alone or in combination with low dosage of LD offered superior long term behavioral protection against PD, when matched with LD alone. Although striatal DA levels are not rescued, the protective effect of CaN inhibition is mainly mediated via maintaining the architecture of striatal neurons.

DA as a substrate of MAO can generate ROS [6, 54] and its uncontrolled production may contribute to neurodegeneration. Our findings show that mitoTEMPO can significantly protect against DA toxicity, implying that mitochondrial ROS is an integral part of DA toxicity. If this is the major contributing factor for DA toxicity, other MAO substrates should also demonstrate similar effects. Although Tyr treatment show toxicity, the dose and time to achieve DA like cell death is much higher. Alternatively, overexpression of MAO A enhances Tyr toxicity, supporting that MAO mediated increase in ROS can be a significant contributory factor. However, how much active MAO is required in vivo to replicate this effect is a matter of exploration. Additionally, elevated DA can lead to cell death even when MAO is present at the endogenous level. This raises the possibility that the other factors associated with DA toxicity, may predominantly narrow down the long term therapeutic window for the MAO inhibitors as an adjuvant of LD therapy. On the other hand, DAQ treatment demonstrates cell death similar to DA, depicting DAQ might contribute highly to DA cytotoxicity [9]. Relevance of DAQ in PD is further supported by the study of Carbajal et al. [55]; which demonstrated Tyrosinase overexpression at SN is enough to induce neurodegeneration. Although Tyrosinase expression in human SN is very low, it might be complemented by auto-oxidation mediated DAQ formation, especially when DA production is heightened at the cellular level. Nevertheless, relevance of DAQ in PD requires further evaluation, as factors like site specific availability of Tyrosinase at physiological level, competition with MAO for DA oxidation and DAQ transport inside neurons can collectively determine its contribution towards SN specific neurodegeneration.

Most often than not, stress induced cell death is reflected on mitochondrial shape and dynamics [22, 28]. DRP1 mediated mitochondrial fragmentation and cell death is well correlated, as high level of DRP1 on mitochondrial membrane can form binding foci for apoptotic proteins [37, 56, 57]. Interestingly, p-Ser637DRP1 decrease after DA/DAQ/Tyr treatment, justifying higher levels of DRP1 on mitochondria and thus fragmentation. CaN mediated mitochondrial translocation of DRP1 can be an apoptotic preconditioning and prerequisite for Cytochrome *c* release from the mitochondrial inter membrane space [32, 58]. However, even though CaN activity increases and p-Ser637DRP1 levels decrease at striatum after LD treatment, we could not find any marked neurodegeneration in vivo. We assume that striatum, being a DA rich region of brain, is well capable to handle sub-acute DA increase and its associated effects. Consequences of chronic DA exposure by this LD dose (10 mg/kg) on long term neuronal survival warrants further investigations. Nonetheless, marked change in striatal spine morphology after 12 days of LD treatment is noticeable, although no significant change in spine density is observed.

Spine density and shape of medium spiny neuron is an outcome of combinations of multiple factors and pathways. However, at the very end, F-Actin in spine head highly determines spine shape and stability [59–61]. As F-Actin formation from G-actin is ATP dependent [62], functional mitochondria distribution along the dendritic shaft (if not in spine head) is required. Both F-Actin stabilization and dendritic mitochondrial distribution can be regulated by CaN [59, 63, 64]. In the current context, nigrostriatal connections remained intact after LD treatment. Thus, there is a limited possibility of LD associated increased striatal extracellular glutamate to destabilize F-Actin and shift the balance towards intermediate (filiform) spine formation [65]. As previously observed, we also assume that mitochondrial fragmentation via CaN-DRP1 axis and increased neuronal activity due to LD treatment might have played a significant part to alter this striatal spine morphology [60, 66]. It is important to mention here that although in cultured neurons, where cell to cell communications are very discrete, DRP1 inactivation decreases spine density [66]. However, complete or partial ablation of DRP1 in vivo, does not lead to any such alterations [67]. On the contrary, partial ablation can be protective against spine loss in a diseased state [68]. This could be due to persistence of synapses which is known to support mature spines.

As dendritic spines represent connectivity, it is a reliable and easier parameter to correlate between animal behavior and intensity of area specific neuronal damage. However, as multiple factors are involved (for example: neurotransmitter release, synaptic vesicle recycling, receptor density, receptor super-sensitivity and proximity between different receptors), neither spine density nor neurotransmitter level (DA in current context) solely can explain behavioral outcomes. In our study, although striatal DA levels in PD mice are not improved by CaN inhibition, dendritic spine density at striatum might have contributed to behavioral recovery. We also found protection in terms of TH positivity at SN or striatum. This raised the possibility that intact striatal projections of DAergic neurons, even at low DA levels, could contribute to protect the spine density and thus behavior. Interestingly, blocking CaN interaction via PxIxIT domain cannot resolve behavioral complexities or dendritic spine density in PD mice; even though DA, TH-positive SN neurons and striatal projections remain protected. Strikingly, the limited neuroprotective effect of VIVIT peptide disappeared when LD is co-treated, resembling retrograde DAergic neurodegeneration. As CaN substrates with PxIxIT docking site include NFAT family proteins [39], we speculate that neuroinflammation might play a role in the current context [69, 70] and thus VIVIT treatment can protect neurons by blocking NFAT activation. As VIVIT is ineffective for CaN-DRP1 axis, it cannot offer the same effect any further when LD is administered in the PD model. The other aspect of MPTP toxicity which may have remained uncovered by VIVIT peptide is the involvement of glutamate. Previous studies have demonstrated that LD can increase extracellular glutamate at striatum when DAergic neurons degenerate [59, 65]. As glutamate is known to destabilize Actin polymerization and decrease spine formation via CaN regulated Slingshot-Cofilin pathway [64], VIVIT might have remained ineffective to protect the mature spines. However, further study is required to delineate such interplay between LD therapy, glutamate, DA release and neuroinflammation during PD progression.

To determine how long CaN inhibition can offer this protection in presence of LD, a different model has to be employed as some of the aspects of MPTP induced PD reverses with time [47, 71]. One limitation of our study is it does not explain why striatal DA levels remain low in FK-506 or LD + FK-506 treated PD mice. Possible explanations include the effect of MPTP on TH activity inhibition [72] and the failure to maintain reserve pool of synaptic vesicles. Previous studies which explored the role of mitochondria and its distribution in neurons, demonstrated that mitochondria (and thus ATP) are required for maintaining the reserve pool of synaptic neurotransmitter vesicles, even though basal synaptic activity remain uninfluenced in absence of active mitochondria [73, 74]. The reserved synaptic vesicles are more relevant for intense or tetanic stimulus [74]. This may partially explain the swim deficiency of FK-506 or LD + FK-506 treated PD mice in our study, even when their akinesia and cataleptic behaviors are attenuated. Our study also does not nullify the possibility that the protective effect of CaN –DRP1 inhibition on behavior might have been influenced by the other parts of basal ganglia or other neurotransmitters.

## Conclusion

In this study, we have established the relevance of the CaN-DRP1 axis in DA toxicity and its implication in delaying the decline of PD-associated behavior or neuro-anatomical aberrations. Previous research has extensively linked neurotoxin-induced sporadic models of PD with DRP1 (64–69), highlighting the importance of addressing mitochondrial irregularities in the early stages, especially when the chosen treatment -LD itself may affect DRP1 localization. FK-506/tacrolimus, a well-tolerated CaN inhibitor currently used therapeutically, shows promise for repurposing against PD progression. However, considering the potential complications associated with long-term FK-506 usage, it is crucial to explore innovative options for specifically inhibiting the DRP1-CaN association. It is important to note that our study does not rule out the possibility of cell type-specific off-target effects and potential complications that may arise from the long-term use of FK-506. Additionally, we acknowledge that there are limitations to the PD model employed in our study, highlighting the need for further validation in different PD models.

## Methods

### Animal treatment

Male C57BL/6 mice are treated with MPTP (30 mg/kg, i.p., Sigma) twice, 16 h apart. FK-506 (1 mg/kg, Panacea Biotech Ltd, India) and/or l-DOPA (5 or 10 mg/kg; carbidopa 4:1, Sun pharma laboratories, India), 1 h apart, are given by gavage for next 5 or 12 days (once daily). Cell permeable PVEET/PVIVIT peptide (H-YGRKKRRQRRR-AAMAGPPHIVEETGPHVI-NH2 and H-YGRKKRRQRRR-AAMAGPHPVIVITGPHEE-NH2 respectively, Abclonal Inc.) [75] are administered subcutaneously for 12 days (0.5 mg/kg, once daily) [76].

### Plasmids and cell line

MAO A plasmid is a kind gift from Dr. Nina Kaludercic (University of Padova, Padova). DRP1 WT/KO MEF cells, DRP1 and CaN mutant plasmids were kindly provided by Dr. Elena Ziviani (University of Padova, Padova). Mito-GFP was kindly provided by Dr. S.N. Bhattacharya (CSIR-Indian Institute of Chemical Biology, Kolkata).

### Cell culture, treatment and toxicity assays

For our study, we employed human midbrain dopaminergic neuronal cell line SH-SY5Y and mouse embryonic fibroblast (MEF). These cell lines are widely utilized to investigate the impact of dopamine and identify crucial mechanisms implicated in the development of Parkinson's disease [77–79]. SH-SY5Y and MEF cell line is maintained in Dulbecco's Modified Eagle’s Medium (10% heat-inactivated fetal bovine serum and 0.1% penicillin/streptomycin, Gibco) at 37 °C in a humidified 5% CO_2_ incubator. Cell transfections are performed using Lipofectamine 2000 (Invitrogen) following the manufacturer’s protocol for 1 µl/µg plasmid DNA.

DA (100–300 µM, SRL, India) is treated for 24 h. Tyr (250–1000 µM, SRL, India) is treated for 16 h/24/48 h. In case of 48 h treatment, media with Tyr is changed after 24 h. Pargyline (100 µM, Sigma), NAC (500 µM, Sigma), mitoTEMPO (10 µM, Sigma), VIVIT (2 µM) or INCA-6 (10 µM, Abcam) is administered 30 min before DA/Tyr. To determine the effect of DAQ, DA (300 µM) was incubated for 30 min with Tyrosinase (20 µ/100 µl, SRL, India) at 37 °C and then the mixture was administered to the cells. Alternatively DA and Tyrosinase is simultaneously administered to the cell culture medium [9].

For Trypan Blue exclusion assay, after the treatment a small volume of cell suspension is mixed with equal volume of trypan blue (Gibco), afterwards viable cells are counted using Countess II FL automated cell counter (Invitrogen). For PI stain, cells are incubated with Hoechst-33342 (1 µg/ml, Invitrogen) and PI (10 µg/ml, SRL, India). Images are taken under fluorescence microscope and cells showing PI in nucleus are counted.

### Mitochondrial morphology and Cytochrome *c* distribution analysis

To analyze mitochondrial morphology after the treatment, live cells are imaged for mitoGFP using a confocal microscope (Zeiss, LSM980). Alternatively, 4% PFA fixed cells were immune-labeled for ATP5a and/or Cytochrome *c* (1:200, Abcam) and appropriate Alexa fluor (1:250, Invitrogen) tagged secondary antibody is used. We employed freely available Fiji plugin Mitomorph to identify filamentous, rod shaped or punctate mitochondria. First the region of interest was cropped from the entire image and the background was subtracted. Threshold was applied to include all mitochondria in a cell and the image is auto analyzed by the plugin [80]. We used JACOP plugin to determine Mander’s coefficient for ATP5a and Cytochrome *c* colocalization.

### SDS-PAGE and immunoblotting

Protein lysate from whole cell or isolated mitochondria are prepared and immunoblotting is done following standard protocol as described previously [81]. The following antibodies were used: Mfn2 (Abcam, 1: 1000), Actin (Abcam, 1:6000), Mfn1 (Millipore, 1:1000), OPA1 (Abcam, 1: 2000), p-ser637DRP1 (Abcam, 1:1000), DRP1 (Abcam, 1: 1000), p-ser616 DRP1 (Cell signaling Technology, 1: 1000), ATP5a (Abcam, 1: 2000). Please see the supplementary table 1 for further details.

### Calcineurin activity assay

CaN activity is determined by following the protocol as mentioned in the kit supplied by Abcam.

### Cytosolic calcium measurement

Cells are washed in HBSS post-treatment and Fluo4-AM solution (Invitrogen, 1 µM) was then loaded as mentioned by the manufacturer. After washing with calcium free HBSS, images are taken using fluorescence microscope (EVOS, Thermo). Signal intensity is measured by utilizing ImageJ.

### Animal behavioral tests

Akinesia and catalepsy are analyzed as described previously [42]. In brief, each animal is acclimatized on an elevated wooden platform (30 cm) for few minutes. Then the latency (in sec) to move all four limbs is noted. For catalepsy, mice are placed on a flat surface and acclimatized for few minutes. After that both forelimbs are kept on a wooden platform (3 cm high) and latency in seconds to move the fore limbs from the wooden platform to the flat surface is noted.

Swim-test is done by placing each animal in water tubs (40 cm length × 25 cm width × 16 cm height), keeping the water depth at 12 cm and the temperature is maintained at 27 ± 2 °C. For next 10 min swim score is noted down as described previously [47].

### Immunohistochemistry

Immunohistochemistry is done as stated earlier [82] for 20 µm striatal/SN sections. Secondary Anti-rabbit antibody-HRP (1:200, Biobharati, India) is used to tag rabbit anti Tyrosine hydroxylase (TH) antibody (1:200, Abcam); 3,3′-Diaminobenzidine tetrahydrochloride (1 mg/2 ml, SRL, India) + H_2_O_2_ (1%) is used to develop the signal. Relevant sections which are known to demonstrate TH depletion after MPTP [83] are taken to count TH positive neurons at SN. Intensity of TH staining at striatum is quantified by using ImageJ.

### Striatal dopamine measurement

Striatal tissue is prepared for DA level measurement following the protocol stated as earlier [84]. Sample is injected into a HPLC system (Thermo ultimate 3000) through a Rheodyne injector, equipped with a glassy carbon electrochemical detector. C18 ion-pair reverse-phase analytical column (5 µm, 4.6 × 150 mm, Acclaim 120) is used to separate the DA at 0.6 ml/min flow rate of mobile phase (8.65 mM heptane sulphonic acid, 0.27 mM EDTA, 13% acetonitrile, 0.43% triethylamine, 0.32% phosphoric acid). DA amount is determined by comparing with a known concentration of standard.

### Golgi staining

Brain samples are preserved in Golgi-Cox staining solution (5 volume parts of 5% potassium dichromate, and 5% mercuric chloride, 4 volume parts of 5% of potassium chromate) [85]. After 21d tissue is transferred to cryoprotectant solution (20% sucrose, 15% glycerol). 60 µm sections are obtained on slide and developed with 20% ammonia solution. Images are acquired by using a confocal microscope (Zeiss, LSM980). Z-stack images of a single neuron are obtained to visualize the nature of neuronal spines. Spines were classified based on their morphology (mushroom, filiform and budding) and were counted from each dendrite of a neuron. Total length of the individual dendrite was noted down. Average total spine density (mushroom + filiform + budding/dendrite length) for each neuron was calculated and represented as spine/µm dendrite.

### Statistics

We used student’s t-test and one way or two way ANOVA followed by Tuckey’s or Dunett’s multiple comparison test for statistical significance, as mentioned in the figure legends. In all cases, results are provided as mean ± SEM. Significance level and number of sample are mentioned wherever relevant.

### Animal ethics

Experiments on animal were accomplished following national guidelines on the “Care and Use of Animals in Scientific Research,” formed by the Committee for the Purpose of Control and Supervision of Experiments on Animals (CPCSEA), Animal Welfare Division, Ministry of Environment and Forests, Govt. of India, and is acknowledged by the animal ethics committee of CSIR-Indian Institute of Chemical Biology, Kolkata, India. C57BL/6 male mice of 8–10 weeks (25–30 g body weight) were kept under regular housing conditions (at 22 ± 2 °C temperature, 60 ± 5% humidity, 12 h of light–dark cycle) and ad libitum food/water were given. Specifications of the items used for this study are provided in additional file 8

## Supplementary Information

**Additional file 1: Figure S1.** MAO A expression in SH-SY5Y cells. SH-SY5Y cells are transfected with MAO A-His plasmid and the expression level is confirmed after 48 h by immunoblotting. Actin is used as a loading control. The immunoblot provided is representative of three different experiments.

**Additional file 2: Figure S2.** Effect of Cyclosporin A (CsA), FK-506 and mitoTEMPO treatment on mitochondrial fragmentation induced by dopamine (DA), tyramine (Tyr) or dopaquinone (DAQ). **(A)** SH-SY5Y cells are treated with DA (200 µM) or DA + CsA (2 µM) as mentioned for 16 h and ATP5a is immunostained to monitor mitochondrial morphology. Mitochondrial morphology is classified as mentioned previously. (B) SH-SY5Y cells are treated with Tyr or DAQ (± FK-506 or mitoTEMPO as mentioned). Images of mitochondria are captured after immunostaining for ATP5a and (C) mitochondrial morphology is analysed. Scale bar: 10 µm. N = 3, at least 30 cells are considered for the analysis. *P ≤ 0.05, **P ≤ 0.01, ***P ≤ 0.001, when compared to the control. ##P ≤ 0.01 when compared to DA treated group. Bar graphs represent mean ± SEM. P values are calculated by one way ANOVA followed by Tukey’s multiple comparison test.

**Additional file 3: Figure S3.** Analysis of DA induced cell death in DRP1 WT or knockout MEF cells. WT or DRP1 KO MEF cell are treated with DA (200 µM) for 24 h and cell death is monitored by trypan blue or propidium iodide (PI) staining. N = 3. **P ≤ 0.01, ***P ≤ 0.001, **** P ≤ 0.0001 when compared to WT group; ####P ≤ 0.0001, when compared to WT + DA and @@ ≤ 0.01 when compared to DRP1 KO cells. Bar graphs represent mean ± SEM. P values are calculated by one way ANOVA followed by Tukey’s multiple comparison test.

**Additional file 4: Figure S4.** Effect of VIVIT or INCA-6 treatment against dopamine (DA) induced mitochondrial fragmentation or cell death. (**A**) SH-SY5Y cells are pretreated with either VIVIT peptide (2 µM) or INCA-6 (10 µM). DA (200 µM) is treated for 16 h to monitor mitochondrial morphology. Images demonstrate immunostaining for ATP5a. The experiment was repeated 3 times. Scale bar 10 µM. (**B**) SH-SY5Y cells are treated as mentioned. 300 µM DA is treated to induce cell death. Cell death is monitored by counting trypan blue or propidium iodide (PI) positive cells. N = 3. **P ≤ 0.01, ***P ≤ 0.001, **** P ≤ 0.0001 when compared to WT control. Bar graphs represent mean ± SEM. P values are calculated by one way ANOVA followed by Tukey’s multiple comparison test.

**Additional file 5: Figure S5.** Assessment of L-DOPA (LD) and FK-506 treatment on Calcineurin (CaN) activity, cell survival and neuronal spine density. (**A and B**) Mice are treated with L-DOPA (5 or 10 mg/kg, LD5 and LD10 respectively, gavage) or FK-506 (gavage) for 12 days and CaN activity is measured. Control mice received equal amount of vehicle. N = 3. *P ≤ 0.05, **P ≤ 0.01 as compared to control. Student’s t test. (**C**) After 5d treatment, animal striatum is processed for immunoblotting and level of p-Ser637DRP1, total DRP1 and Actin was measured. N = 3, *P ≤ 0.05 as compared to control group. One way ANOVA followed by Dunnett’s multiple comparison test. (**D**) Animal brain striatum is processed to detect TUNEL positive cells (red nuclei) after 12d of LD treatment. Cell nuclei are counter stained by DAPI. Scale bar- 10 µm. At least 3 animal brain striatum are analyzed. (**E and F**) After LD treatment, SN or striatal brain sections (**E** and **F** respectively) are immunostained for Tyrosine hydroxylase. Image magnification is as mentioned in Fig. 5a. Images are representative of at least 3 different experiments. (**G and H**) After 12d of treatment, as mentioned, striatum is processed for Golgi-Cox staining. Images demonstrate representative part of dendrite from the neuron (**G**, inset). Total spine or spine density based on morphology is represented by the bar graphs (± SEM). Striatum from 4 animals are taken and 4–5 neurons from each brain are utilized for the analysis. Scale bar 10 µm. Bar graphs represent mean ± SEM.

**Additional file 6: Figure S6.** Calcineurin activity and p-Ser637DRP1 levels in MPTP treated mice striatum. (**A**) Calcineruin activity is measured on 8th day from striatal protein homogenate. LD (10 mg/kg) is treated for 5 days. N = 3–4. (**B**) Total and p-Ser637DRP1 protein levels are measured form total striatal protein homogenate. Immunoblot is representative of 3 different experiments. *P ≤ 0.05; **P ≤ 0.01 as compared to control group. One way ANOVA followed by Dunnett’s multiple comparison test. Bar graphs represent mean ± SEM.

**Additional file 7: Figure S7.** Outcome of VEET peptide treatment on PD associated motor activity, dopaminerigic neuronal population or striatal dendritic spine density. (**A and B**) VEET peptide is treated (0.5 mg/kg, sub- cutaneous, for 12 days) to the MPTP administered mice and their akinesia (**A**) or cataleptic behavior (**B**) is analyzed. N = 4. **P ≤ 0.01, ***P ≤ 0.001, **** P ≤ 0.0001 when compared to control. **(C)** After the treatment, as mentioned above, striatal dopamine level is quantified using HPLC based method. N = 4, **P ≤ 0.01 when compared to control. (**D**) After the treatment period, as mentioned, images are taken for Tyrosine hydroxylase positive neurons at substantia nigra. Image magnification is as mentioned in Fig. (5A). At least 3 animal brains are utilized for this experiment. (**E**) After the treatment period, striatal tissue was processed for Golgi-Cox staining. Images exhibit representative part of dendrite from the neuron at inset. Total spine density is represented by the bar graph. 4–5 animal striatum is taken and 4–5 neurons each brain are utilized for the analysis. **** P ≤ 0.0001 when compared to control. Bar graphs represent mean ± SEM. One way ANOVA followed by Dunnett’s multiple comparison test.

**Additional file 8.** Items used with specifications.

## Data Availability

Data and materials can be available on request.

## Abbreviations

MPTP: 1-Methyl-4-phenyl-1,2,3,6-tetrahydropyridine
CaN: Calcineurin
DAQ: DA-quinone
DA: Dopamine
DAergic: Dopaminergic
DRP1: Dynamin related protein 1
GFP: Green fluorescence protein
LD: L-DOPA
MFN: Mitofusin
MAO: Monoamine oxidase
MEF: Mouse embryonic fibroblast
OPA1: Optic atrophy type 1
PFA: Paraformaldehyde
PD: Parkinson’s disease
PI: Propidium iodide
SN: Substantia nigra
Tyr: Tyramine
TH: Tyrosine hydroxylase
