# Supplementary figures and images for: Calcineurin inhibition protects against dopamine toxicity and attenuates behavioral decline in a Parkinson’s disease model
Source: Cell Biosci. 2023 Aug 1;13:140. doi: 10.1186/s13578-023-01068-6 (PMC10394860; doi:10.1186/s13578-023-01068-6)

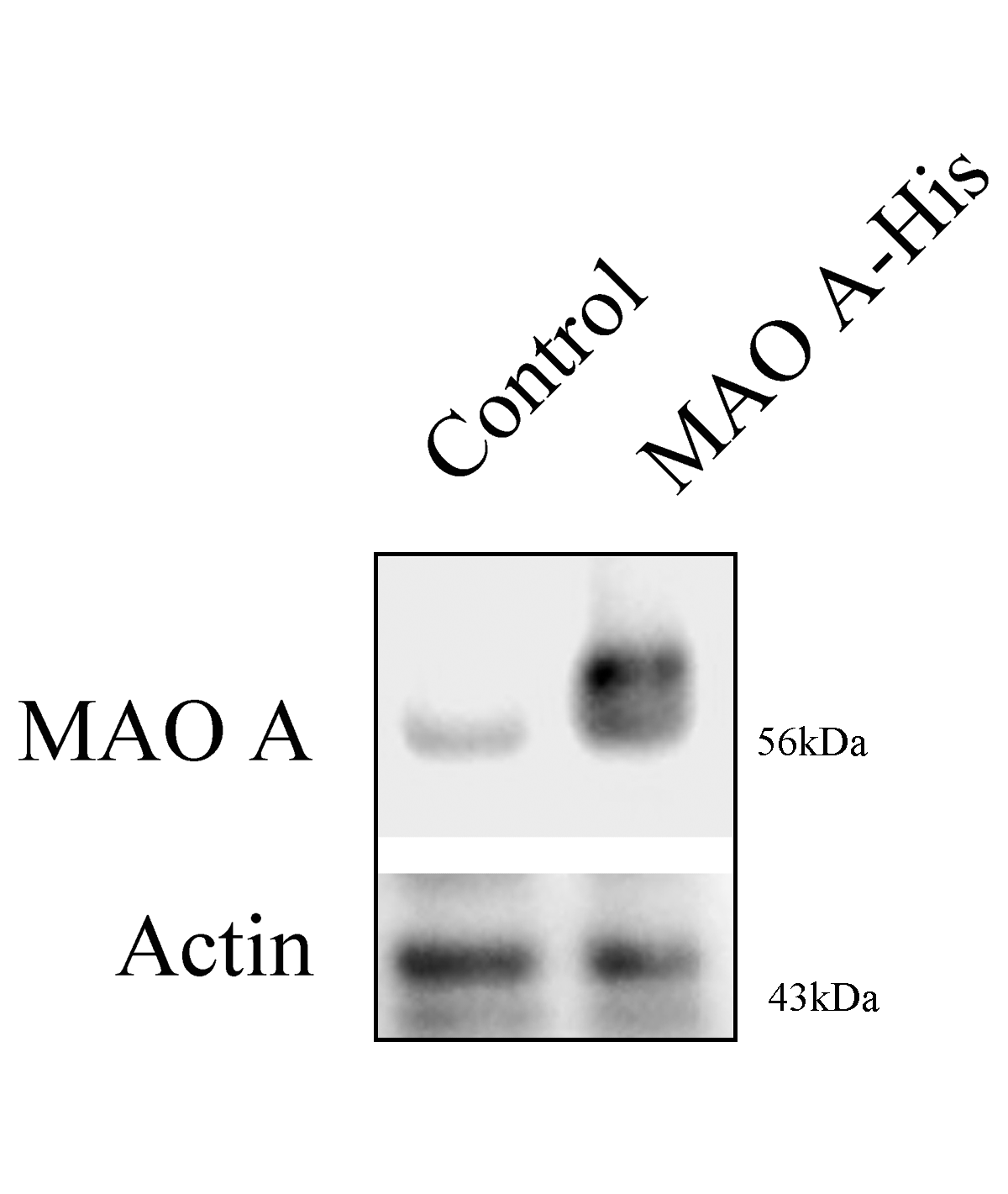

Supplement: Supplementary file 1 — Additional file 1: Figure S1. MAO A expression in SH-SY5Y cells. SH-SY5Y cells are transfected with MAO A-His plasmid and the expression level is confirmed after 48 h by immunoblotting. Actin is used as a loading control. The immunoblot provided is representative of three different experiments. [file 13578_2023_1068_MOESM1_ESM.tif]

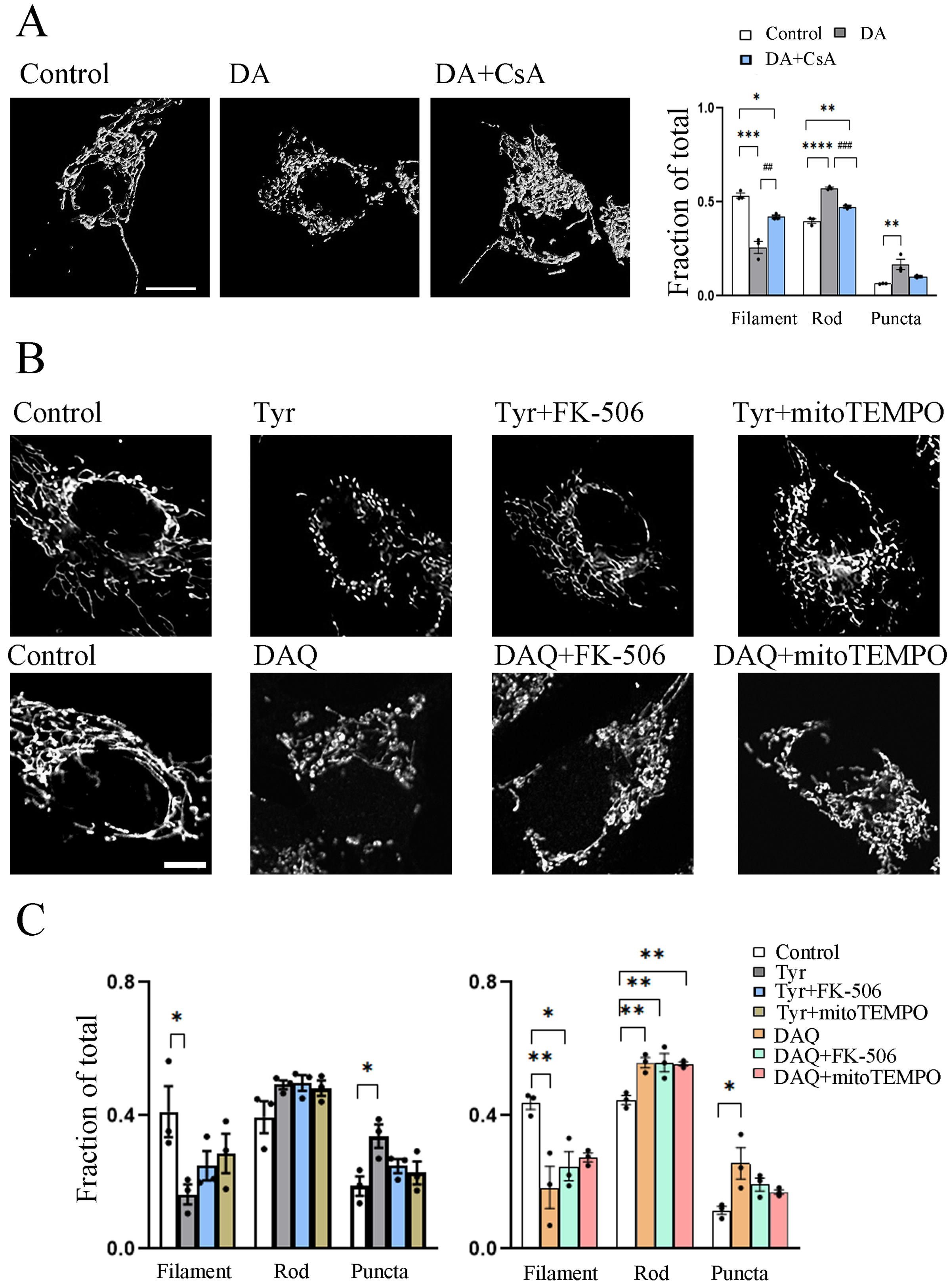

Supplement: Supplementary file 2 — Additional file 2: Figure S2. Effect of Cyclosporin A (CsA), FK-506 and mitoTEMPO treatment on mitochondrial fragmentation induced by dopamine (DA), tyramine (Tyr) or dopaquinone (DAQ). (A) SH-SY5Y cells are treated with DA (200 µM) or DA + CsA (2 µM) as mentioned for 16 h and ATP5a is immunostained to monitor mitochondrial morphology. Mitochondrial morphology is classified as mentioned previously. (B) SH-SY5Y cells are treated with Tyr or DAQ (± FK-506 or mitoTEMPO as mentioned). Images of mitochondria are captured after immunostaining for ATP5a and (C) mitochondrial morphology is analysed. Scale bar: 10 µm. N = 3, at least 30 cells are considered for the analysis. *P ≤ 0.05, **P ≤ 0.01, ***P ≤ 0.001, when compared to the control. ##P ≤ 0.01 when compared to DA treated group. Bar graphs represent mean ± SEM. P values are calculated by one way ANOVA followed by Tukey’s multiple comparison test. [file 13578_2023_1068_MOESM2_ESM.tif]

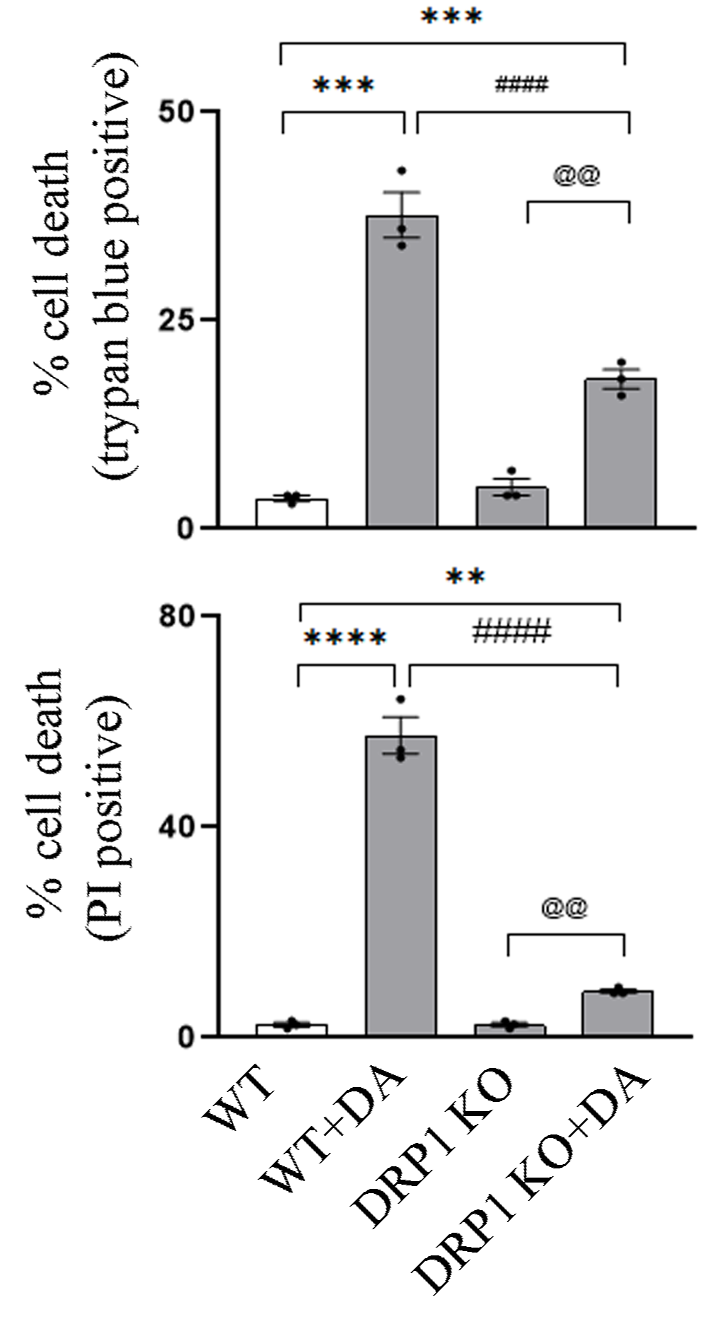

Supplement: Supplementary file 3 — Additional file 3: Figure S3. Analysis of DA induced cell death in DRP1 WT or knockout MEF cells. WT or DRP1 KO MEF cell are treated with DA (200 µM) for 24 h and cell death is monitored by trypan blue or propidium iodide (PI) staining. N = 3. **P ≤ 0.01, ***P ≤ 0.001, **** P ≤ 0.0001 when compared to WT group; ####P ≤ 0.0001, when compared to WT + DA and @@ ≤ 0.01 when compared to DRP1 KO cells. Bar graphs represent mean ± SEM. P values are calculated by one way ANOVA followed by Tukey’s multiple comparison test. [file 13578_2023_1068_MOESM3_ESM.tif]

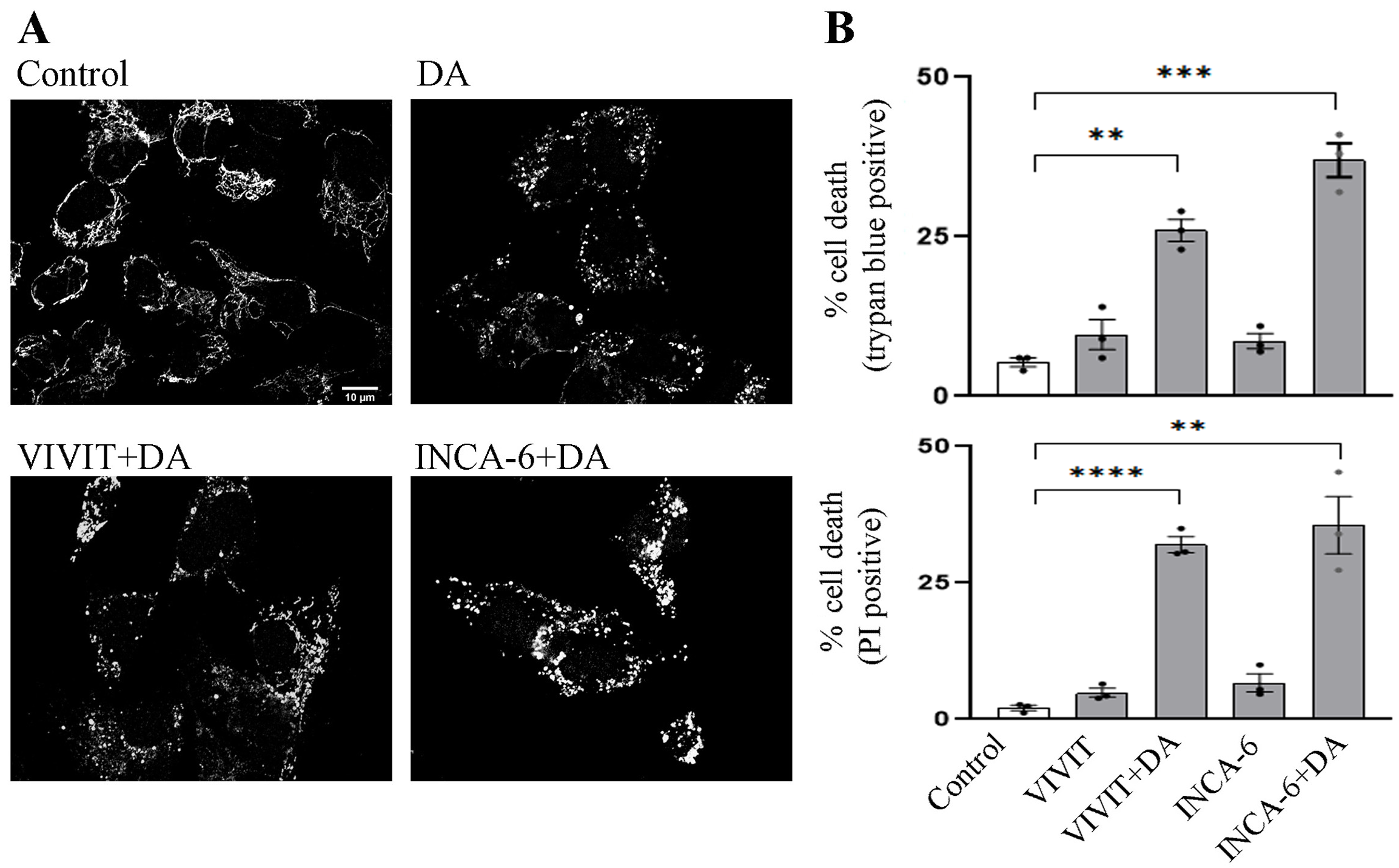

Supplement: Supplementary file 4 — Additional file 4: Figure S4. Effect of VIVIT or INCA-6 treatment against dopamine (DA) induced mitochondrial fragmentation or cell death. (A) SH-SY5Y cells are pretreated with either VIVIT peptide (2 µM) or INCA-6 (10 µM). DA (200 µM) is treated for 16 h to monitor mitochondrial morphology. Images demonstrate immunostaining for ATP5a. The experiment was repeated 3 times. Scale bar 10 µM. (B) SH-SY5Y cells are treated as mentioned. 300 µM DA is treated to induce cell death. Cell death is monitored by counting trypan blue or propidium iodide (PI) positive cells. N = 3. **P ≤ 0.01, ***P ≤ 0.001, **** P ≤ 0.0001 when compared to WT control. Bar graphs represent mean ± SEM. P values are calculated by one way ANOVA followed by Tukey’s multiple comparison test. [file 13578_2023_1068_MOESM4_ESM.tif]

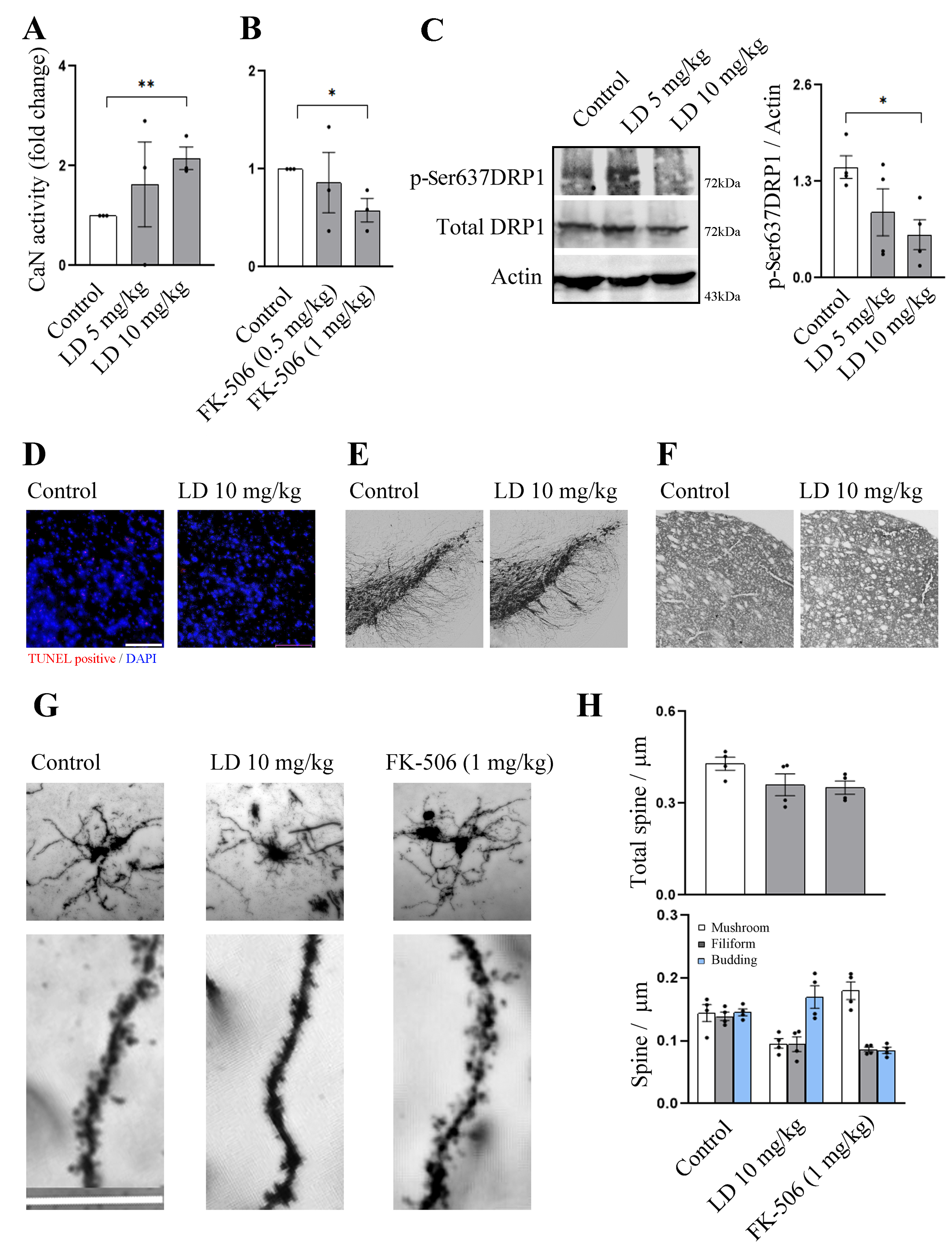

Supplement: Supplementary file 5 — Additional file 5: Figure S5. Assessment of L-DOPA (LD) and FK-506 treatment on Calcineurin (CaN) activity, cell survival and neuronal spine density. (A and B) Mice are treated with L-DOPA (5 or 10 mg/kg, LD5 and LD10 respectively, gavage) or FK-506 (gavage) for 12 days and CaN activity is measured. Control mice received equal amount of vehicle. N = 3. *P ≤ 0.05, **P ≤ 0.01 as compared to control. Student’s t test. (C) After 5d treatment, animal striatum is processed for immunoblotting and level of p-Ser637DRP1, total DRP1 and Actin was measured. N = 3, *P ≤ 0.05 as compared to control group. One way ANOVA followed by Dunnett’s multiple comparison test. (D) Animal brain striatum is processed to detect TUNEL positive cells (red nuclei) after 12d of LD treatment. Cell nuclei are counter stained by DAPI. Scale bar- 10 µm. At least 3 animal brain striatum are analyzed. (E and F) After LD treatment, SN or striatal brain sections (E and F respectively) are immunostained for Tyrosine hydroxylase. Image magnification is as mentioned in Fig. 5a. Images are representative of at least 3 different experiments. (G and H) After 12d of treatment, as mentioned, striatum is processed for Golgi-Cox staining. Images demonstrate representative part of dendrite from the neuron (G, inset). Total spine or spine density based on morphology is represented by the bar graphs (± SEM). Striatum from 4 animals are taken and 4–5 neurons from each brain are utilized for the analysis. Scale bar 10 µm. Bar graphs represent mean ± SEM. [file 13578_2023_1068_MOESM5_ESM.tif]

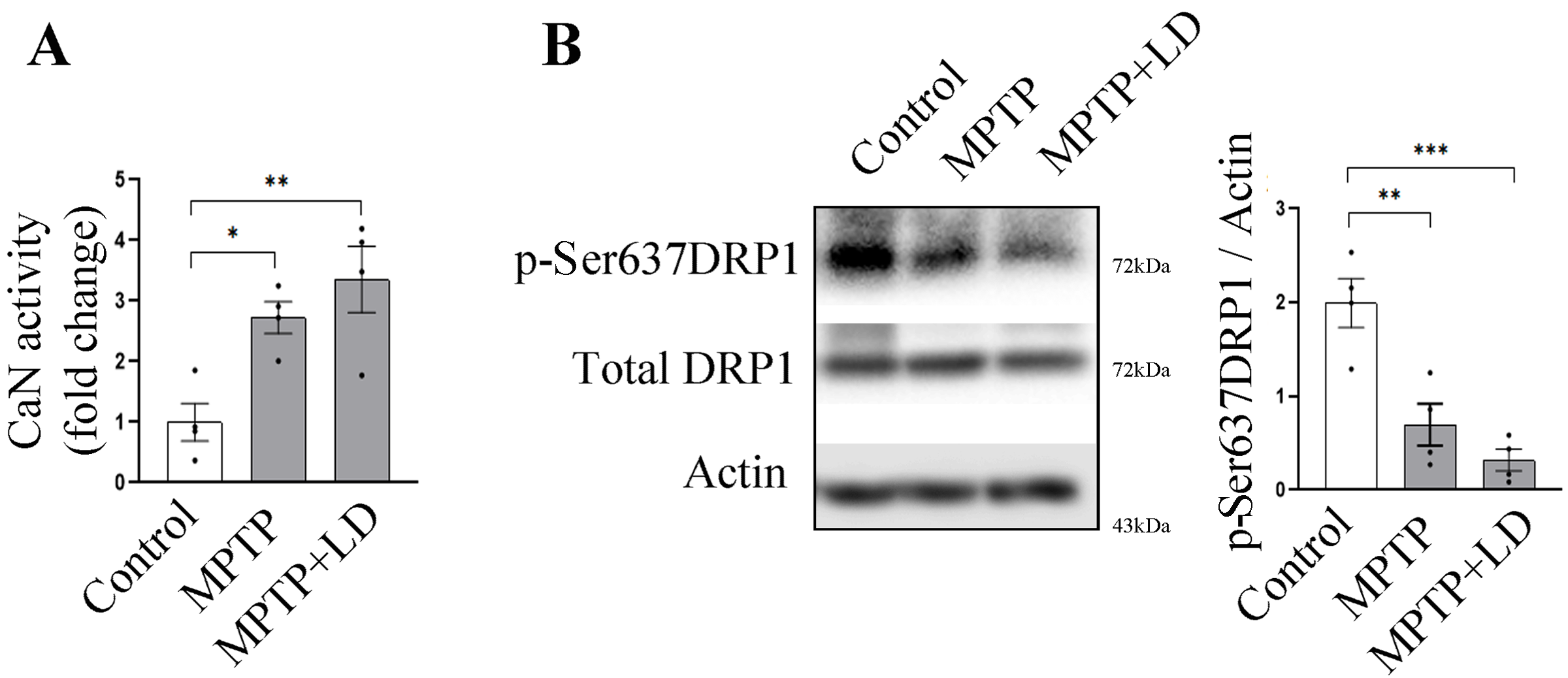

Supplement: Supplementary file 6 — Additional file 6: Figure S6. Calcineurin activity and p-Ser637DRP1 levels in MPTP treated mice striatum. (A) Calcineruin activity is measured on 8th day from striatal protein homogenate. LD (10 mg/kg) is treated for 5 days. N = 3–4. (B) Total and p-Ser637DRP1 protein levels are measured form total striatal protein homogenate. Immunoblot is representative of 3 different experiments. *P ≤ 0.05; **P ≤ 0.01 as compared to control group. One way ANOVA followed by Dunnett’s multiple comparison test. Bar graphs represent mean ± SEM. [file 13578_2023_1068_MOESM6_ESM.tif]

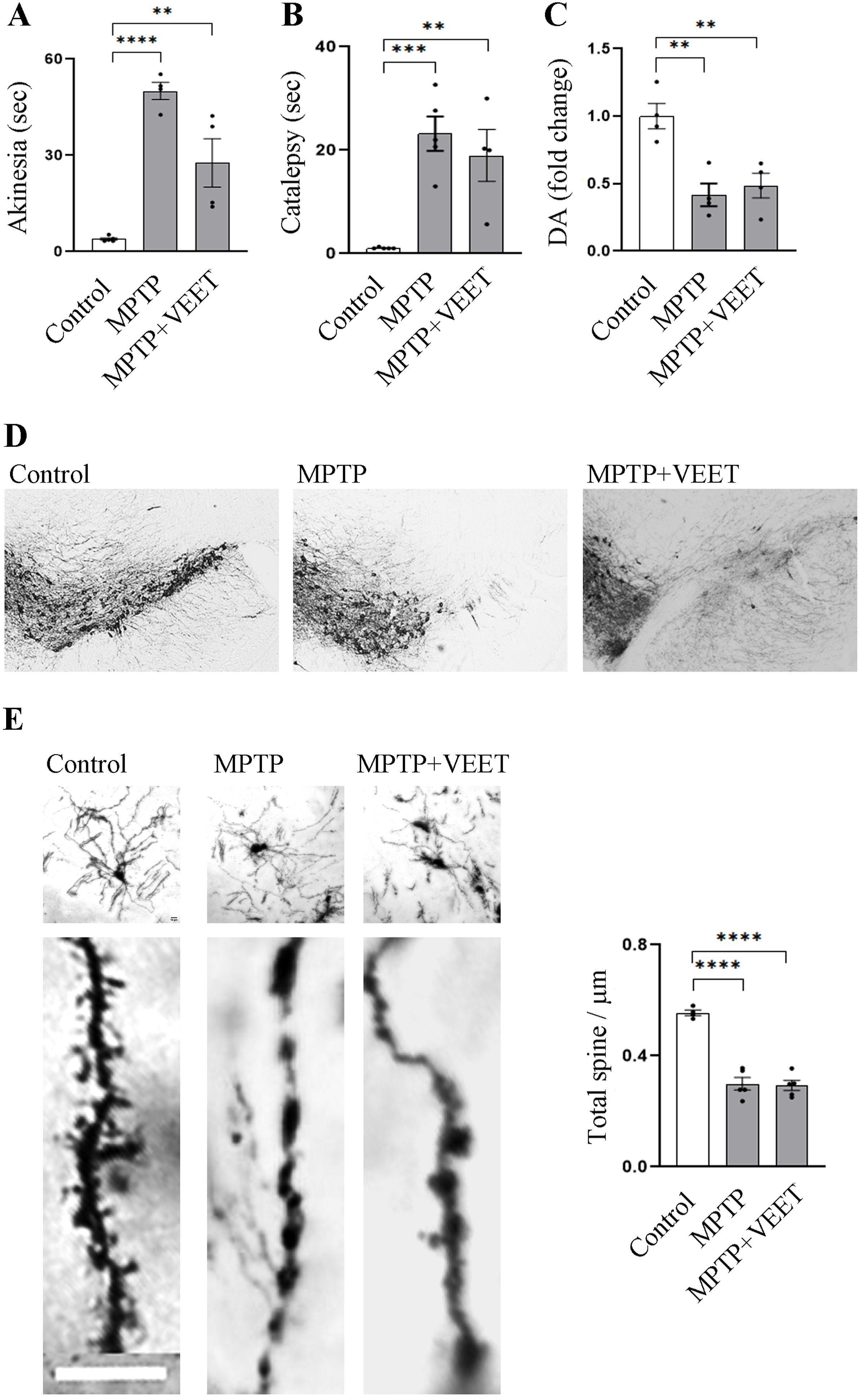

Supplement: Supplementary file 7 — Additional file 7: Figure S7. Outcome of VEET peptide treatment on PD associated motor activity, dopaminerigic neuronal population or striatal dendritic spine density. (A and B) VEET peptide is treated (0.5 mg/kg, sub- cutaneous, for 12 days) to the MPTP administered mice and their akinesia (A) or cataleptic behavior (B) is analyzed. N = 4. **P ≤ 0.01, ***P ≤ 0.001, **** P ≤ 0.0001 when compared to control. (C) After the treatment, as mentioned above, striatal dopamine level is quantified using HPLC based method. N = 4, **P ≤ 0.01 when compared to control. (D) After the treatment period, as mentioned, images are taken for Tyrosine hydroxylase positive neurons at substantia nigra. Image magnification is as mentioned in Fig. (5A). At least 3 animal brains are utilized for this experiment. (E) After the treatment period, striatal tissue was processed for Golgi-Cox staining. Images exhibit representative part of dendrite from the neuron at inset. Total spine density is represented by the bar graph. 4–5 animal striatum is taken and 4–5 neurons each brain are utilized for the analysis. **** P ≤ 0.0001 when compared to control. Bar graphs represent mean ± SEM. One way ANOVA followed by Dunnett’s multiple comparison test. [file 13578_2023_1068_MOESM7_ESM.tif]
