# Supplementary material for: Calcineurin inhibition protects against dopamine toxicity and attenuates behavioral decline in a Parkinson’s disease model
Source: Cell Biosci. 2023 Aug 1;13:140. doi: 10.1186/s13578-023-01068-6 (PMC10394860; doi:10.1186/s13578-023-01068-6)
Supplement: Supplementary file 8 — Additional file 8. Items used with specifications. [file 13578_2023_1068_MOESM8_ESM.docx]

| **Item** | **Manufacturer** | **Cat no** |
| --- | --- | --- |
| MPTP hydrochloride | Sigma Aldrich | M0896 |
| FK-506 | Sigma Aldrich | F4679 |
| PVEET / PVIVIT peptide | Abclonal Inc | Custom made |
| Dulbecco’s Modified Eagle Medium (DMEM), High glucose | Gibco | 12800-017 |
| Fetal Bovine Serum (FBS) | Gibco | 10270-106 |
| Penicillin-Streptomycin (PS) | Gibco | 15070-063 |
| Lipofectamine 2000 | Invitrogen | 11668019 |
| Dopamine | Sisco Research Laboratories Pvt. Ltd. | 45462 |
| Tyramine | Sisco Research Laboratories Pvt. Ltd. | 33541 |
| Pargyline hydrochloride | Sigma Aldrich | P8013 |
| MitoTEMPO | Sigma Aldrich | SML0737 |
| N-Acetyl-L-cysteine (NAC) | Sigma Aldrich | A7250 |
| Tyrosinase | Sisco Research Laboratories Pvt. Ltd. | 94870 |
| Trypan blue | Gibco | 15250061 |
| Hoechst-33342 | Invitrogen | H1399 |
| Propidium iodide | Sisco Research Laboratories Pvt. Ltd. | 11195 |
| Alexa Fluor 488 goat anti-rabbit | Invitrogen | A11008 |
| Alexa Fluor 568 goat anti-mouse | Invitrogen | A11004 |
| ProLong Gold antifade reagent | Invitrogen | P36941 |
| Cellular Calcineurin phosphatase activity assay kit | Abcam | ab139464 |
| Hanks′ Balanced Salt solution | Sigma Aldrich | H9394 |
| Fluo-4, AM | Invitrogen | F14201 |
| 3,3-Diaminobenzidine Tetrahydrochloride Hydrate (DAB) | Sisco Research Laboratories Pvt. Ltd. | 17076 |
| Heptane sulphonic acid | Sisco Research Laboratories Pvt. Ltd. | 94373 |
| EDTA | Sisco Research Laboratories Pvt. Ltd. | 43272 |
| Acetonitrile (HPLC grade) | Sisco Research Laboratories Pvt. Ltd. | 58209 |
| Trielhylamine | Sisco Research Laboratories Pvt. Ltd. | 67352 |
| Phosphoric acid | Qualigens | 29215 |
| INCA-6 | Abcam | ab145864 |
| Potassium dichromate | Merk Millipore | 104864 |
| Potassium chromate | Merk Millipore | 104952 |
| Mercuric chloride | Sisco Research Laboratories Pvt. Ltd. | SRL-25699 |
| Cyclosporine A | Sigma Aldrich | C3662 |
| Anti-ATP5A antibody | Abcam | ab14748 |
| Anti-Cytochrome C antibody | Abcam | ab133504 |
| Anti-MFN1 antibody | Merck | ABC41 |
| Anti-MFN2 antibody | Abcam | ab56889 |
| Anti-OPA1 antibody | Abcam | ab42364 |
| Anti-DRP1 antibody | Abcam | ab56788 |
| Anti-DRP1 (phospho S637) antibody | Abcam | ab193216 |
| Anti-DRP1 (phospho S637) antibody | Sigma Aldrich | SAB4301399 |
| Anti-DRP1 (phospho S616) antibody | Cell signalling technology | 3455 |
| Anti-Actin antibody | Abcam | ab179467 |
| Anti-Tyrosine Hydroxylase antibody | Abcam | ab112 |
| Anti-rabbit antibody-HRP | Biobharati | BB-SAB01B |
| Anti-mouse antibody-HRP | Biobharati | BB-SAB02B |
